# Assessment of Regional Variability in COVID-19 Outcomes Among Patients With Cancer in the United States

**DOI:** 10.1001/jamanetworkopen.2021.42046

**Published:** 2022-01-04

**Authors:** Jessica E. Hawley, Tianyi Sun, David D. Chism, Narjust Duma, Julie C. Fu, Na Tosha N. Gatson, Sanjay Mishra, Ryan H. Nguyen, Sonya A. Reid, Oscar K. Serrano, Sunny R. K. Singh, Neeta K. Venepalli, Ziad Bakouny, Babar Bashir, Mehmet A. Bilen, Paolo F. Caimi, Toni K. Choueiri, Scott J. Dawsey, Leslie A. Fecher, Daniel B. Flora, Christopher R. Friese, Michael J. Glover, Cyndi J. Gonzalez, Sharad Goyal, Thorvardur R. Halfdanarson, Dawn L. Hershman, Hina Khan, Chris Labaki, Mark A. Lewis, Rana R. McKay, Ian Messing, Nathan A. Pennell, Matthew Puc, Deepak Ravindranathan, Terence D. Rhodes, Andrea V. Rivera, John Roller, Gary K. Schwartz, Sumit A. Shah, Justin A. Shaya, Mitrianna Streckfuss, Michael A. Thompson, Elizabeth M. Wulff-Burchfield, Zhuoer Xie, Peter Paul Yu, Jeremy L. Warner, Dimpy P. Shah, Benjamin French, Clara Hwang

**Affiliations:** 1 Herbert Irving Comprehensive Cancer Center at Columbia University, New York, New York; 2 now with Division of Oncology, University of Washington/Fred Hutchinson Cancer Research Center, Seattle; 3 Vanderbilt University Medical Center, Nashville, Tennessee; 4 Thompson Cancer Survival Center, Knoxville, Tennessee; 5 University of Wisconsin Carbone Cancer Center, Madison; 6 Tufts Medical Center Cancer Center, Boston and Stoneham, Massachusetts; 7 Geisinger Health System, Danville, Pennsylvania; 8 Banner MD Anderson Cancer Center, Gilbert, Arizona; 9 University of Illinois Hospital & Health Sciences System, Chicago; 10 Hartford HealthCare Cancer Institute, Hartford, Connecticut; 11 Henry Ford Cancer Institute, Henry Ford Hospital, Detroit, Michigan; 12 University of North Carolina, Lineberger Cancer Center, Chapel Hill; 13 Dana-Farber Cancer Institute, Boston, Massachusetts; 14 Sidney Kimmel Cancer Center at Thomas Jefferson University, Philadelphia, Pennsylvania; 15 Winship Cancer Institute of Emory University, Atlanta, Georgia; 16 Case Comprehensive Cancer Center at Case Western Reserve University/University Hospitals, Cleveland, Ohio; 17 Cleveland Clinic Taussig Cancer Institute, Cleveland, Ohio; 18 University of Michigan Rogel Cancer Center, Ann Arbor; 19 St Elizabeth Healthcare, Edgewood, Kentucky; 20 Stanford Cancer Institute at Stanford University, Palo Alto, California; 21 George Washington University, Washington, DC; 22 Mayo Clinic, Rochester, Minnesota; 23 Brown University and Lifespan Cancer Institute, Providence, Rhode Island; 24 Intermountain Healthcare, Salt Lake City, Utah; 25 University of California, San Diego; 26 Virtua Health, Marlton, New Jersey; 27 University of Kansas Medical Center, Kansas City; 28 Advocate Aurora Health, Milwaukee, Wisconsin; 29 Mays Cancer Center at UT Health San Antonio MD Anderson Cancer Center, San Antonio, Texas

## Abstract

**Importance:**

The COVID-19 pandemic has had a distinct spatiotemporal pattern in the United States. Patients with cancer are at higher risk of severe complications from COVID-19, but it is not well known whether COVID-19 outcomes in this patient population were associated with geography.

**Objective:**

To quantify spatiotemporal variation in COVID-19 outcomes among patients with cancer.

**Design, Setting, and Participants:**

This registry-based retrospective cohort study included patients with a historical diagnosis of invasive malignant neoplasm and laboratory-confirmed SARS-CoV-2 infection between March and November 2020. Data were collected from cancer care delivery centers in the United States.

**Exposures:**

Patient residence was categorized into 9 US census divisions. Cancer center characteristics included academic or community classification, rural-urban continuum code (RUCC), and social vulnerability index.

**Main Outcomes and Measures:**

The primary outcome was 30-day all-cause mortality. The secondary composite outcome consisted of receipt of mechanical ventilation, intensive care unit admission, and all-cause death. Multilevel mixed-effects models estimated associations of center-level and census division–level exposures with outcomes after adjustment for patient-level risk factors and quantified variation in adjusted outcomes across centers, census divisions, and calendar time.

**Results:**

Data for 4749 patients (median [IQR] age, 66 [56-76] years; 2439 [51.4%] female individuals, 1079 [22.7%] non-Hispanic Black individuals, and 690 [14.5%] Hispanic individuals) were reported from 83 centers in the Northeast (1564 patients [32.9%]), Midwest (1638 [34.5%]), South (894 [18.8%]), and West (653 [13.8%]). After adjustment for patient characteristics, including month of COVID-19 diagnosis, estimated 30-day mortality rates ranged from 5.2% to 26.6% across centers. Patients from centers located in metropolitan areas with population less than 250 000 (RUCC 3) had lower odds of 30-day mortality compared with patients from centers in metropolitan areas with population at least 1 million (RUCC 1) (adjusted odds ratio [aOR], 0.31; 95% CI, 0.11-0.84). The type of center was not significantly associated with primary or secondary outcomes. There were no statistically significant differences in outcome rates across the 9 census divisions, but adjusted mortality rates significantly improved over time (eg, September to November vs March to May: aOR, 0.32; 95% CI, 0.17-0.58).

**Conclusions and Relevance:**

In this registry-based cohort study, significant differences in COVID-19 outcomes across US census divisions were not observed. However, substantial heterogeneity in COVID-19 outcomes across cancer care delivery centers was found. Attention to implementing standardized guidelines for the care of patients with cancer and COVID-19 could improve outcomes for these vulnerable patients.

## Key Points

**Question:**

To what extent are spatiotemporal trends in the COVID-19 pandemic in the United States associated with outcomes for patients with cancer infected with SARS-CoV-2?

**Findings:**

This cohort study of 4749 patients with cancer and COVID-19 found no significant differences in outcomes across the 9 US census divisions. Overall, outcomes significantly improved between March and December 2020, and treatment at cancer centers in less densely populated counties was associated with better outcomes.

**Meaning:**

These findings suggest that understanding the heterogeneity in COVID-19 outcomes between cancer centers could guide resource allocation and help the oncology community improve COVID-19 outcomes for this patient population.

## Teaser

This cohort study examines spatiotemporal variation in COVID-19 outcomes among patients with cancer in the United States.

## Introduction

While the novel SARS-CoV-2 virus has affected populations on all continents except Antarctica, the United States has experienced unique spatiotemporal trends in COVID-19 cases. In March 2020, COVID-19 began to affect major urban centers in the United States, with the identification of cases in Seattle, Washington; New York City; Detroit, Michigan; and New Orleans, Louisiana.^1^ Throughout 2020, COVID-19 outbreaks spread from the Northeast to the South and West before becoming widespread across the nation. Understanding whether patient outcomes vary by region is important, as it may guide further resource allocation, assistance, and vaccination efforts. It is particularly important for patients with cancer who are at high risk of major complications due to COVID-19,^2,3,4,5^ as such understanding may assist oncologic societies, cancer center leadership and administration, and clinical oncology health care authorities formulate effective measures and outreach to combat the ongoing pandemic.

The COVID-19 and Cancer Consortium (CCC19) is, to our knowledge, the largest multicenter registry study examining the clinical characteristics, course of illness, and outcomes among patients with cancer and COVID-19.^2,3^ In this study, we quantified differences in clinical outcomes across US census geographic divisions among patients with cancer and COVID-19. We hypothesized that major clinical outcomes of COVID-19 (receipt of mechanical ventilation, intensive care unit [ICU] admission, and death) would significantly vary across the US given the fluctuating case rates, resource burdens, and potential rationing of care. We also summarized outcome rates across cancer centers and determined whether characteristics of those centers were associated with outcomes.

## Methods

### Study Cohort

The CCC19 registry (NCT04354701) collects detailed information on patients with cancer and COVID-19.^3,6,7^ Data on patients with presumptive COVID-19 or laboratory-confirmed SARS-CoV-2 who have a current or prior diagnosis of invasive malignant neoplasm are extracted from electronic health records by investigators at participating institutions^7^ and managed using a REDCap database hosted at Vanderbilt University Medical Center (eAppendix 1 in Supplement 1).^8^ Follow-up data are collected at 30, 90, and 180 days after COVID-19 diagnosis. The CCC19 data dictionary is available online.^9^ We conducted a retrospective cohort study of patients reported to the CCC19 registry before November 30, 2020, with follow up through December 31, 2020. The analysis was limited to patients with a laboratory-confirmed diagnosis of SARS-CoV-2 by polymerase chain reaction and/or serology (presumptive cases were excluded), with 30-day follow-up and high-quality data (score <5 according to our previously published metric^10^). The analyses were further restricted to patients known to reside in the United States; reports submitted anonymously, for which a center was not identified, were excluded (eFigure in Supplement 1). This study was either exempt from institutional review board (IRB) review or approved by IRBs at all participating centers, per individual institutional policy. The Vanderbilt University institutional review board determined that informed consent was not required, and all data were deidentified. Reporting of results follows the Strengthening the Reporting of Observational Studies in Epidemiology (STROBE) reporting guidelines.^11^

### Outcomes

The primary outcome was 30-day all-cause mortality. The secondary outcome was a composite binary outcome consisting of receipt of mechanical ventilation, ICU admission, and all-cause death. This secondary composite outcome was assessed over the patients’ total follow-up period and was selected because of interest in capturing overall health care burden more generally.

### Region and Center Variables

We defined US geographical regions according to the US Census Bureau designation of regions and divisions. Each of the 4 regions (Northeast, Midwest, South, and West) consist of 2 or 3 divisions. Each of the 9 divisions (New England [Connecticut, Maine, Massachusetts, New Hampshire, Rhode Island, and Vermont], Middle Atlantic [New Jersey, New York, and Pennsylvania], East North Central [Indiana, Illinois, Michigan, Ohio, and Wisconsin], West North Central [Iowa, Kansas, Minnesota, Missouri, Nebraska, North Dakota, and South Dakota], South Atlantic [Delaware, Washington, DC, Florida, Georgia, Maryland, North Carolina, South Carolina, Virginia, and West Virginia], East South Central [Alabama, Kentucky, Mississippi, and Tennessee], West South Central [Arkansas, Louisiana, Oklahoma, and Texas], Mountain [Arizona, Colorado, Idaho, New Mexico, Montana, Utah, Nevada, and Wyoming], Pacific [Alaska, California, Hawaii, Oregon, and Washington]) consist of several states. Patients were classified according to their reported state of residence. The mean rate of SARS-CoV-2 diagnosis per season was computed for each division using an aggregate of states’ new COVID-19 case rate per 1 million^12^ and the US Census Bureau’s estimated state populations for 2019 (eTable 1 in Supplement 1).^13^

We also considered how clinical outcomes across regions might be associated with social factors and levels of urbanization for cancer centers within those regions. We therefore used 2 metrics commonly used in the public health and emergency response planning fields: the US Centers for Disease Control and Prevention’s Social Vulnerability Index (SVI)^14^ and the US Department of Agriculture’s Economic Research Service’s 2013 Rural-Urban Continuum Code (RUCC). The SVI indicates the relative vulnerability of every US census tract (or county). SVI ranks census tracts on 15 social factors in 4 domains: socioeconomic status, household composition and disability, minority status and language, and housing and transportation. An overall percentile rank (0-1) for the county in which each center is located was used in this analysis. An SVI ranking of 0.85 indicates that 85% of tracts (or counties) in the state or nation are less vulnerable than the tract of interest and that 15% of tracts (or counties) in the state or nation are more vulnerable. In this study, centers were located only in RUCC 1, 2, and 3, in which 1 indicates a metropolitan area with at least 1 million population; 2, metropolitan area with between 250 000 and 1 million population; and 3, metropolitan area with fewer than 250 000 million population (eMethods in Supplement 1).^15^ We also classified centers as either academic or community. In the case of a participating institution having more than 1 site contributing patient cases, center-level data from the flagship center was used.

### Patient Characteristics

Patient-level factors included age, sex, race and ethnicity (Black, Hispanic, White, other [American Indian or Alaska Native, Asian, and Native Hawaiian or other Pacific Islander], and missing or unknown), smoking status, obesity, comorbidities (cardiovascular disease, pulmonary disease, renal disease, diabetes), Eastern Cooperative Oncology Group (ECOG) performance status, type of cancer, cancer status (remission or no evidence of disease, active and stable or responding to therapy, active and progressing, or unknown), timing of anticancer therapy relative to COVID-19 diagnosis, modality of active anticancer therapy (chemotherapy, targeted therapy, endocrine therapy, immunotherapy, or surgery/radiation), anti–COVID-19 treatments (including remdesivir, hydroxychloroquine, and corticosteroids), and month of COVID-19 diagnosis, which was categorized to capture the spread and seasonality of the pandemic across the US as spring (March to May 2020), summer (June to August 2020), and fall (September to November 2020).^2^ Models were adjusted for race and ethnicity given their different distributions in US census divisions and known racial and ethnic disparities in clinical care and outcomes of patients with SARS-CoV-2.^16^

### Statistical Analysis

A statistical analysis plan was prespecified prior to analysis (eAppendix 2 in Supplement 1), which commenced on February 10, 2021. Standard descriptive statistics were used to summarize patient-level characteristics and outcome rates according to the region in which the patient resided. Center-level characteristics were summarized according to the region in which the center was located. For parsimony of presentation, these summaries are presented across 4 regions. Unadjusted outcome rates were calculated for each center and division over the entire study period; division-level rates were also summarized by calendar time.

Multivariable generalized linear mixed-effects models (with a logit link and center-level random intercepts) were used to analyze the primary (30-day mortality) and secondary (composite) binary outcomes. For the composite outcome, the model included an offset for (log) follow-up time. A series of models were fit. The first included patient-level characteristics, including month of COVID-19 diagnosis, to calculate adjusted outcome rates across centers.^17^ The second model added center-level characteristics to estimate their associations with outcomes after adjusting for patient-level characteristics. The third model added division-level covariates to quantify differences across divisions after adjusting for patient- and center-level characteristics. Because of the complexity and computational demands of these models, as well as limitations on model degrees of freedom, we did not adjust for timing or modality of anticancer therapies. Results are presented as adjusted odds ratios (aORs) and 95% CIs.

Multiple imputation (10 iterations; missingness rates were <5%) using additive regression, bootstrapping, and predictive mean matching was used to impute missing and unknown data. Unknown ECOG performance status and unknown cancer status were not imputed and included as unknown categories. Imputation was performed on the full data set of 5083 participants prior to exclusions. All analyses were performed in R version 4.0.4. (R Project for Statistical Computing), including the lme4 extension package. Statistical significance was set at *P* < .05, and all tests were 2-tailed.

## Results

### Patient Characteristics

In total, 4749 patients with cancer and COVID-19 were included in our analyses (eFigure in Supplement 1). The median (IQR) age was 66 (56-76) years, and 2439 (51.4%) were female patients (Table 1). There were 1079 (22.7%) Black patients, and 690 (14.9%) Hispanic patients. Overall, 1564 (32.9%) resided in the Northeast, 1638 (34.5%) in the Midwest, 653 (18.8%) in the South, and 4749 (13.8%) in the West. More Black patients were reported in the Midwest (454 [27.7%]) and South (300 [33.6%]) compared with the Northeast (297 [19.0%]) and West (28 [4.3%]); substantially more Hispanic patients were reported in the West (202 [30.9%]) compared with other regions. Approximately half of patients in the Northeast and Midwest were classified as current or former smokers compared with the South (329 [36.8%]) and West (223 [34.2%]). There were no substantial differences in ECOG performance status, type of cancer, cancer status, or anticancer therapies across regions. However, we observed differences in the use of COVID-19 treatments across regions. Remdesivir was used to a greater extent in the West (87 [13.3%]) compared with other regions (range, 114 [7.0%] to 95 [10.6%]), while hydroxychloroquine was used to a greater extent in the Northeast (371 [23.7%]) compared with other regions (range, 19 [2.9%] to 293 [17.9%]). The spatiotemporal pattern of COVID-19 cases across the United States was reflected in patients with cancer. In the Northeast, a large majority of cases (1212 [77.5%]) were diagnosed March to May. From June to August, the South and the West experienced their highest proportion of cases (436 [48.8%] and 335 [51.3%], respectively). The West had the highest proportion of cases from September to November (121 [18.5%]).

**Table 1.** Demographic and Clinical Characteristics at COVID-19 Diagnosis Among Adults With Cancer, Stratified by US Census Region^a^

Characteristic	No. (%)				
	Northeast	Midwest	South	West	Total
**Patient-level characteristics^b^**					
No.	1564	1638	894	653	4749
Age, median (IQR), y^c^	68.0 (58.0-78.0)	66.0 (56.0-76.0)	64.0 (54.0-73.0)	63.0 (52.0-73.0)	66.0 (56.0-76.0)
Sex					
Female	816 (52.2)	836 (51.0)	443 (49.6)	344 (52.7)	2439 (51.4)
Male	748 (47.8)	799 (48.8)	451 (50.4)	308 (47.2)	2306 (48.6)
Missing or unknown	0	3 (0.2)	0	1 (0.2)	4 (0.1)
Race and ethnicity					
Black	297 (19.0)	454 (27.7)	300 (33.6)	28 (4.3)	1079 (22.7)
Hispanic	304 (19.4)	71 (4.3)	113 (12.6)	202 (30.9)	690 (14.5)
White	819 (52.4)	957 (58.4)	405 (45.3)	292 (44.7)	2473 (52.1)
Other^d^	131 (8.4)	120 (7.3)	60 (6.7)	95 (14.5)	406 (8.5)
Missing or unknown	13 (0.8)	36 (2.2)	16 (1.8)	36 (5.5)	101 (2.1)
Smoking status					
Never	791 (50.6)	803 (49.0)	521 (58.3)	416 (63.7)	2531 (53.3)
Ever	730 (46.7)	801 (48.9)	329 (36.8)	223 (34.2)	2083 (43.9)
Missing or unknown	43 (2.7)	34 (2.1)	44 (4.9)	14 (2.1)	135 (2.8)
Obesity^e^					
No	1011 (64.6)	875 (53.4)	538 (60.2)	400 (61.3)	2824 (59.5)
Yes	543 (34.7)	750 (45.8)	346 (38.7)	251 (38.4)	1890 (39.8)
Missing or unknown	10 (0.6)	13 (0.8)	10 (1.1)	2 (0.3)	35 (0.7)
Comorbid conditions^f^					
Cardiovascular	550 (35.2)	546 (33.3)	261 (29.2)	157 (24.0)	1514 (31.9)
Pulmonary	390 (24.9)	366 (22.3)	173 (19.4)	99 (15.2)	1028 (21.6)
Kidney disease	297 (19.0)	269 (16.4)	150 (16.8)	77 (11.8)	793 (16.7)
Diabetes	464 (29.7)	491 (30.0)	246 (27.5)	150 (23.0)	1351 (28.4)
Missing or unknown	14 (0.9)	18 (1.1)	13 (1.5)	3 (0.5)	48 (1.0)
ECOG performance status					
0	535 (34.2)	608 (37.1)	316 (35.3)	182 (27.9)	1641 (34.6)
1	432 (27.6)	397 (24.2)	266 (29.8)	130 (19.9)	1225 (25.8)
≥2	315 (20.1)	251 (15.3)	107 (12.0)	68 (10.4)	741 (15.6)
Unknown	276 (17.6)	379 (23.1)	204 (22.8)	273 (41.8)	1132 (23.8)
Missing	6 (0.4)	3 (0.2)	1 (0.1)	0	10 (0.2)
Type of cancer^f^					
Solid tumor	1243 (79.5)	1371 (83.7)	694 (77.6)	540 (82.7)	3848 (81.0)
Hematological neoplasm	372 (23.8)	314 (19.2)	230 (25.7)	127 (19.4)	1043 (22.0)
Cancer status					
Remission or no evidence of disease	740 (47.3)	961 (58.7)	440 (49.2)	330 (50.5)	2471 (52.0)
Active and stable or responding	467 (29.9)	400 (24.4)	247 (27.6)	189 (28.9)	1303(27.4)
Active and progressing	217 (13.9)	161 (9.8)	115 (12.9)	70 (10.7)	563 (11.9)
Unknown	137 (8.8)	116 (7.1)	91 (10.2)	64 (9.8)	408 (8.6)
Missing	3 (0.2)	0	1 (0.1)	0	4 (0.1)
Timing of anticancer therapy relative to COVID-19 diagnosis					
Never treated	142 (9.1)	132 (8.1)	65 (7.3)	56 (8.6)	395 (8.3)
0-3 mo before	693 (44.3)	543 (33.2)	400 (44.7)	253 (38.7)	1889 (39.8)
>3 mo before	682 (43.6)	889 (54.3)	387 (43.3)	322 (49.3)	2280 (48.0)
Missing or unknown	47 (3.0)	74 (4.5)	42 (4.7)	22 (3.4)	185 (3.9)
Modality of recent anticancer therapy^f^^,^^g^					
None	852 (54.5)	1062 (64.8)	477 (53.4)	391 (59.9)	2782 (58.6)
Cytotoxic chemotherapy	293 (18.7)	220 (13.4)	163 (18.2)	91 (13.9)	767 (16.2)
Targeted therapy	230 (14.7)	177 (10.8)	163 (18.2)	86 (13.2)	656 (13.8)
Endocrine therapy	169 (10.8)	154 (9.4)	85 (9.5)	61 (9.3)	469 (9.9)
Immunotherapy	91 (5.8)	59 (3.6)	44 (4.9)	45 (6.9)	239 (5.0)
Locoregional therapy	127 (8.1)	142 (8.7)	75 (8.4)	65 (10.0)	409 (8.6)
Other	9 (0.6)	11 (0.7)	6 (0.7)	6 (0.9)	32 (0.7)
Missing or unknown	19 (1.2)	33 (2.0)	17 (1.9)	9 (1.4)	78 (1.6)
Anti–COVID-19 treatments^f^					
None	834 (53.3)	957 (58.4)	547 (61.2)	430 (65.8)	2768 (58.3)
Remdesivir	148 (9.5)	114 (7.0)	95 (10.6)	87 (13.3)	444 (9.3)
Hydroxychloroquine	371 (23.7)	293 (17.9)	93 (10.4)	19 (2.9)	776 (16.3)
Corticosteroids	217 (13.9)	265 (16.2)	147 (16.4)	108 (16.5)	737 (15.5)
Other	431 (27.6)	382 (23.3)	194 (21.7)	123 (18.8)	1130 (23.8)
Missing or unknown	25 (1.6)	67 (4.1)	34 (3.8)	19 (2.9)	145 (3.1)
Month of COVID-19 diagnosis, 2020					
March to May	1212 (77.5)	897 (54.8)	359 (40.2)	197 (30.2)	2665 (56.1)
June to August	206 (13.2)	501 (30.6)	436 (48.8)	335 (51.3)	1478 (31.1)
September to November	138 (8.8)	237 (14.5)	95 (10.6)	121 (18.5)	591 (12.4)
Missing or unknown	8 (0.5)	3 (0.2)	4 (0.4)	0	15 (0.3)
**Center-level characteristics^h^**					
No.	22	23	23	15	83
SVI, median (IQR)	0.537 (0.423-0.738)	0.512 (0.264-0.678)	0.620 (0.449-0.724)	0.487 (0.339-0.635)	0.537 (0.333-0.709)
RUCC^i^					
1	16 (72.7)	18 (78.3)	11 (47.8)	14 (93.3)	59 (71.1)
2	5 (22.7)	1 (4.3)	11 (47.8)	1 (6.7)	18 (21.7)
3	1 (4.5)	4 (17.4)	1 (4.3)	0	6 (7.2)
Type					
Academic	13 (59.1)	15 (65.2)	14 (60.9)	7 (46.7)	49 (59.0)
Community	9 (40.9)	8 (34.8)	9 (39.1)	8 (53.3)	34 (41.0)
Patients, median (IQR), No.^j^	44 (24-105)	55 (36-104)	32 (21-40)	36 (18-73)	36 (25-98)

Abbreviations: ECOG, Eastern Cooperative Oncology Group; RUCC, rural-urban continuum code; SVI, social vulnerability index.

^a^ The missing or unknown category indicates either missingness due to nonresponse for optional survey questions or a response of unknown; an unknown category was provided for all survey questions.

^b^ Patients were grouped into census divisions according to the location of their residence.

^c^ For patients younger than 18 years, age was truncated to 18 years; for patients older than 89 years, age was truncated to 90 years. Truncation was done in concordance with the Health Insurance Portability and Accountability Act of 1996 and to reduce the risk of reidentifiability.

^d^ Other race and ethnicity includes American Indian or Alaska Native, Asian, and Native Hawaiian or other Pacific Islander.

^e^ Patients reported to have obesity or a body mass index (calculated as weight in kilograms divided by height in meters squared) of at least 30.

^f^ Percentages could sum to greater than 100% because categories are not mutually exclusive.

^g^ Within 3 months prior to COVID-19 diagnosis.

^h^ Centers were grouped into census divisions according to the location of the institution.

^i^ RUCC 1 indicates metropolitan with at least 1 million population; RUCC 2, metropolitan area with 250 000 to 1 million population; RUCC 3, metropolitan area with fewer than 250 000 population.

^j^ Number of patients reported to the COVID-19 and Cancer Consortium and included in this analysis.

### Center-Level Characteristics and Outcomes

In total, 83 centers were included in our analysis, spread evenly across regions (Northeast, 22 [26.5%]; Midwest, 23 [27.7%]; South, 23 [26.5%]; and West, 15 [18.1%]) (Table 1). Midwest centers contributed the greatest number of patient reports. Most centers in the West were classified as community based, while most in the Northeast, Midwest, and South were classified as academic. The centers in the South had the highest median (IQR) SVI score (0.620 [0.449-0.724]), while the centers in the West had the lowest median (IQR) SVI score (0.487 [0.339-0.635]); the Northeast and Midwest centers had similar median SVI. Across all regions, centers were located exclusively in metropolitan areas (RUCC 1-3). None of the centers were classified as a nonmetropolitan area (RUCC 4-9). Most centers had RUCC codes of 1 across regions with the exception of the South, which had equal numbers of centers classified by RUCC 1 and RUCC 2 code.

**Figure 1.**
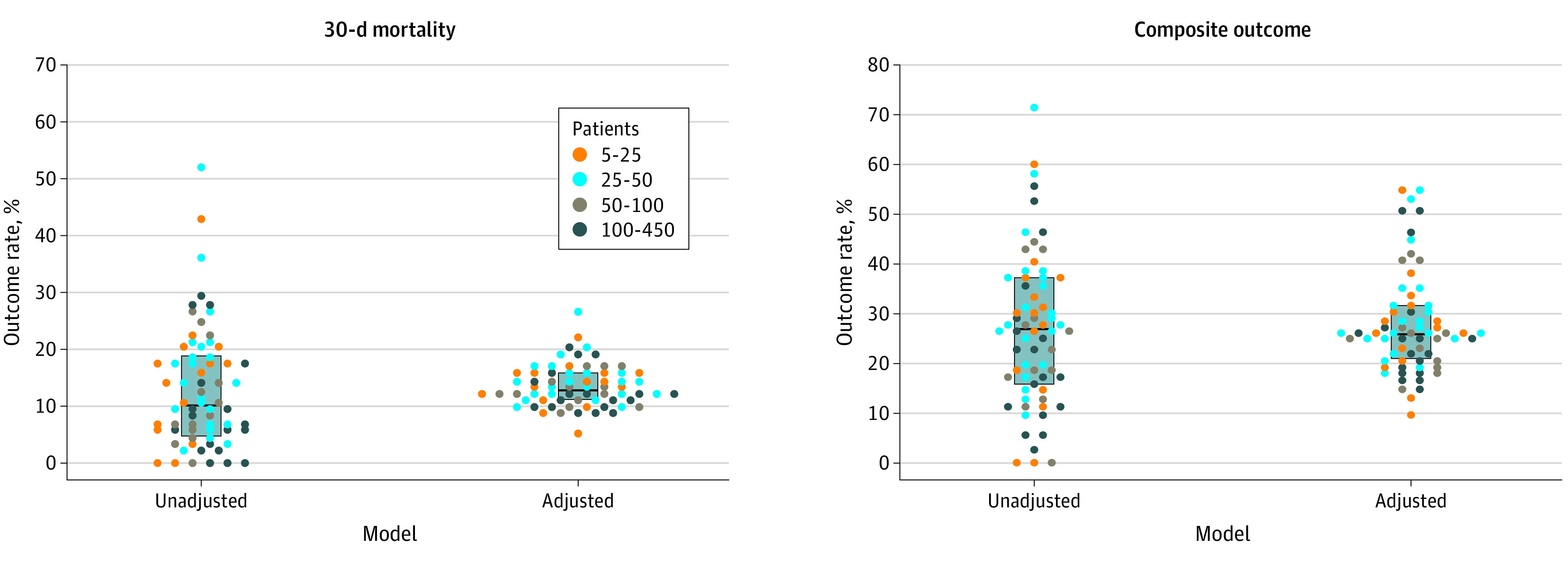
Center-Level Unadjusted and Adjusted Rates of 30-Day All-Cause Mortality and Secondary Composite Outcome (Receipt of Mechanical Ventilation, Intensive Care Unit Admission, and All-Cause Death) Each dot represents a center, colored according to the number of patients reported to the COVID-19 and Cancer Consortium and included in this analysis. Dots are not displayed for 6 centers with fewer than 5 patients. Median and interquartile range are denoted by the center line and box, respectively. Estimated standard deviation of the center-level random intercepts: 0.555 and 0.837 (on the log-odds scale) for 30-day mortality and the composite outcome, respectively.

We observed substantial heterogeneity across centers in unadjusted and adjusted outcome rates (Figure 1). Across centers, the median (IQR) adjusted rate was 12.9% (11.2%-15.9%) for 30-day mortality and 26.1% (20.9%-31.0%) for the composite outcome. Less densely populated areas exhibited a statistically significant association with better outcomes: patients from centers located in metropolitan areas with population of fewer than 250 000 residents (RUCC 3) had lower odds of 30-day mortality compared with patients from centers in metropolitan areas with population of at least 1 million (RUCC 1; aOR, 0.31; 95% CI, 0.11-0.84) (Table 2). To explore this result further, we summarized the initial severity of COVID-19 at presentation by the RUCC classification system. We observed a higher frequency of severe disease (ie, ICU admission indicated, whether or not it occurred) at centers with RUCC 1 and 2 compared with those with RUCC 3 (eTable 2 in Supplement 1).

**Table 2.** Adjusted Associations of Center- and Division-Level Factors With 30-Day All-Cause Mortality and the Composite Outcome

Factor	aOR (95% CI)	
	30-d mortality^a^	Composite outcome^b^
**Center-level factors^c^**		
SVI, per 0.1 unit difference	1.04 (0.95-1.13)	0.98 (0.87-1.09)
RUCC^d^		
1	1 [Reference]	1 [Reference]
2	0.88 (0.56-1.38)	0.87 (0.49-1.52)
3	0.31 (0.11-0.84)	0.26 (0.09-0.75)
Type		
Academic	1 [Reference]	1 [Reference]
Community	1.38 (0.91-2.10)	0.81 (0.48-1.39)
**Division-level factors^e^**		
Mean rate of SARS-CoV-2 diagnosis, per 100 cases per million population	1.01 (0.79-1.31)	0.99 (0.79-1.24)
Census division^f^		
New England	1.36 (0.79-2.34)	1.45 (0.70-3.01)
Middle Atlantic	1.50 (0.85-2.64)	1.28 (0.63-2.58)
East North Central	1 [Reference]	1 [Reference]
West North Central	1.24 (0.60-2.58)	1.38 (0.61-3.13)
South Atlantic	1.36 (0.75-2.49)	1.46 (0.75-2.87)
East South Central	1.36 (0.67-2.76)	1.50 (0.64-3.48)
West South Central	1.78 (0.82-3.85)	1.88 (0.70-5.00)
Mountain	1.24 (0.47-3.26)	1.40 (0.49-4.04)
Pacific	0.83 (0.42-1.66)	0.67 (0.30-1.52)

Abbreviations: aOR, adjusted odds ratio; RUCC, rural-urban continuum code; SVI, social vulnerability index.

^a^ Odds ratios greater than 1 indicate higher odds of 30-day all-cause mortality.

^b^ The composite outcome reflected the occurrence of any of the following: admission to an intensive care unit, receipt of mechanical ventilation, and total all-cause mortality. Analyses of the composite outcome were limited to 4561 patients within nonmissing data. Odds ratios greater than 1 indicate higher odds of admission to an intensive care unit, receipt of mechanical ventilation, or total all-cause mortality.

^c^ Adjusted for age, sex, race and ethnicity, smoking status, obesity, cardiovascular comorbidities, pulmonary comorbidities, kidney disease, diabetes, type of cancer, cancer status, Eastern Cooperative Oncology Group performance status, anti–COVID-19 treatments, and month of COVID-19 diagnosis. All variance inflation factors were less than 5.

^d^ RUCC 1 indicates metropolitan with at least 1 million population; RUCC 2, metropolitan area with 250 000 to 1 million population; RUCC 3, metropolitan area with fewer than 250 000 population.

^e^ Adjusted for age, sex, race and ethnicity, smoking status, obesity, cardiovascular comorbidities, pulmonary comorbidities, kidney disease, diabetes, type of cancer, cancer status, Eastern Cooperative Oncology Group performance status, anti–COVID-19 treatments, month of COVID-19 diagnosis, and all center-level factors. All variance inflation factors were less than 5.

^f^ *P* values for evaluating the null hypothesis of equality in ORs across census divisions (8 *df*): 30-day mortality, .73; composite outcome, .63.

### Division-Level Characteristics and Outcomes

**Figure 2.**
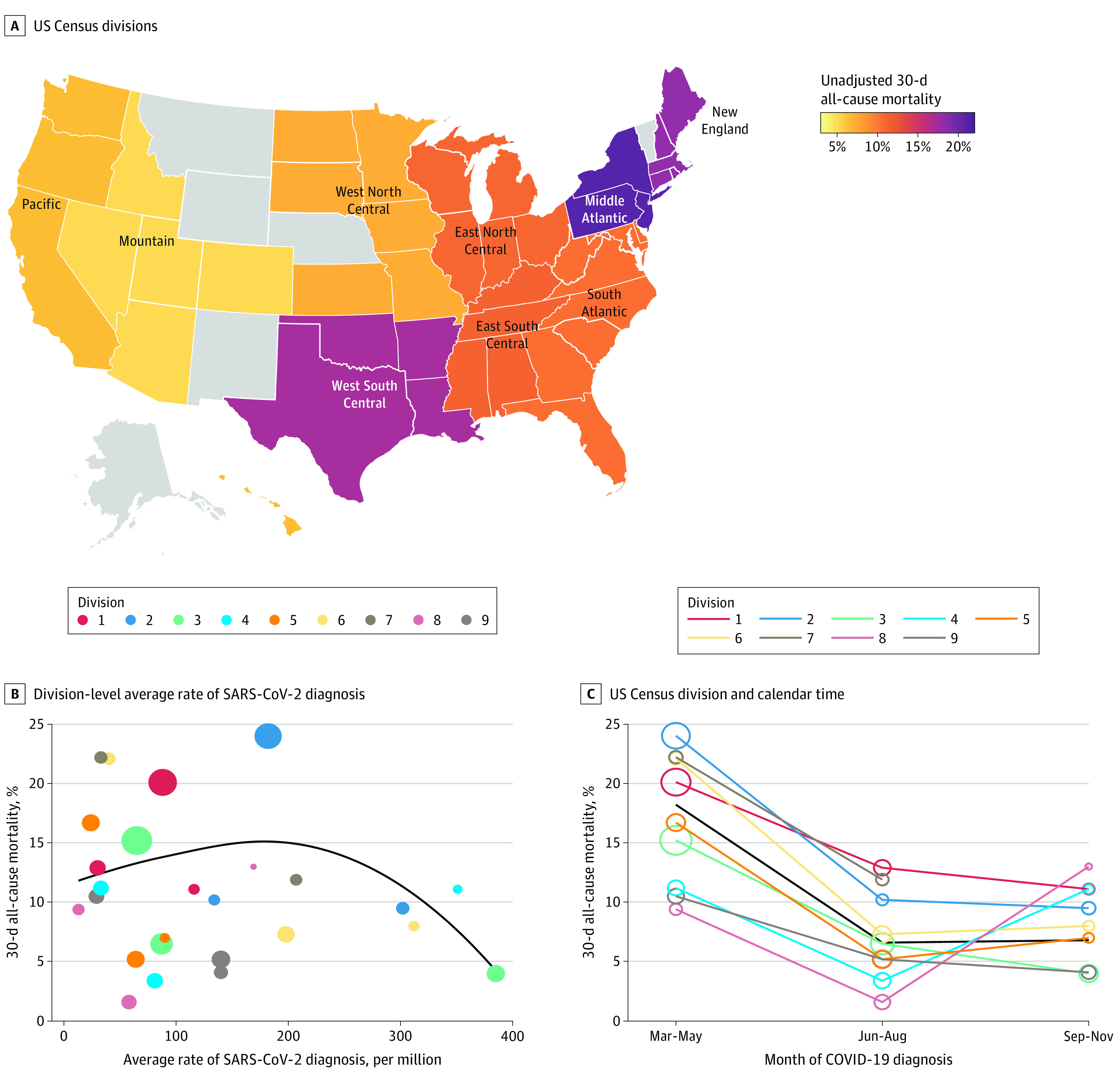
Unadjusted Rates of 30-Day All-Cause Mortality by 9 US Census Divisions, Division-Level Average Rate of SARS-CoV-2 Diagnosis, and US Census Division and Calendar Time A, Gray color indicates that there were no residents of that state reported to the COVID-19 and Cancer Consortium. B and C, Color-coded points represent each US census division. The size of the point is proportional to the number of patients in the COVID-19 and Cancer Consortium who were diagnosed with COVID-19 in that division during each time point. Division 7 (West South Central) is excluded at the third time point because there were fewer than 5 patients. In panel B, the black line is a lowess smoother to represent the average association between the rate of SARS-CoV-2 diagnosis and 30-day mortality; in panel C, the black line represents all divisions. Division 1, New England, includes Connecticut, Maine, Massachusetts, New Hampshire, Rhode Island, and Vermont; division 2, Middle Atlantic, includes New Jersey, New York, and Pennsylvania; division 3, East North Central, includes Indiana, Illinois, Michigan, Ohio, and Wisconsin; division 4, West North Central, includes Iowa, Kansas, Minnesota, Missouri, Nebraska, North Dakota, and South Dakota; division 5, South Atlantic, includes Delaware, Washington, DC, Florida, Georgia, Maryland, North Carolina, South Carolina, Virginia, and West Virginia; division 6, East South Central, includes Alabama, Kentucky, Mississippi, and Tennessee; division 7, West South Central, includes Arkansas, Louisiana, Oklahoma, and Texas; division 8, Mountain, includes Arizona, Colorado, Idaho, New Mexico, Montana, Utah, Nevada, and Wyoming; and division 9, Pacific, includes Alaska, California, Hawaii, Oregon, and Washington.

The Northeast region had the highest 30-day mortality rate (19.6% [306 patients]) compared with the South (12.0% [107 patients]), Midwest (10.4% [171 patients]), and West (6.1% [40 patients]) (Table 3 and Figure 2A). The Northeast also had the highest proportion of unadjusted composite outcome (34.2% [507 patients]) compared with the South (24.2% [211 patients]), Midwest (23.1% [368 patients]), and West (17.9% [110 patients]). This was associated with the fact that centers in the Northeast had the highest proportions of patients admitted to an ICU (14.0% [213 patients]) in our cohort and also the highest total all-cause mortality (25.0% [390 patients]). The centers in the South had the largest proportion of patients receiving mechanical ventilation (18.1% [160 patients]), although proportions were similar in the Northeast (17.8% [265 patients]), Midwest (17.6% [282 patients]) and West (15.2% [95 patients]).

**Table 3.** Outcomes Following COVID-19 Diagnosis Among Adults With Cancer, Stratified by US Census Region

Outcome	Northeast, No./total No. (%)^a^	Midwest, No./total No. (%)^a^	South, No./total No. (%)^a^	West, No./total No. (%)^a^
30-d all-cause mortality^b^	306/1564 (19.6)	171/1638 (10.4)	107/894 (12.0)	40/653 (6.1)
Composite outcome^c^	507/1484 (34.2)	368/1590 (23.1)	211/873 (24.2)	110/614 (17.9)
Total all-cause mortality	390/1561 (25.0)	210/1626 (12.9)	125/885 (14.1)	52/643 (8.1)
Receipt of mechanical ventilation	265/1490 (17.8)	282/1606 (17.6)	160/882 (18.1)	95/624 (15.2)
Admission to an intensive care unit	213/1517 (14.0)	183/1611 (11.4)	90/885 (10.2)	54/621 (8.7)

^a^ Total number of patients are those with nonmissing data.

^b^ Primary outcome.

^c^ Secondary composite outcome that reflected the occurrence of any of the following: admission to an intensive care unit, receipt of mechanical ventilation, and total all-cause mortality.

In adjusted models, there were no statistically significant differences in outcome rates across census divisions (Table 2; eTable 3 in Supplement 1). There was no clear association between the average rate of SARS-CoV-2 diagnosis (per 100 cases per million) and 30-day mortality (Figure 2B), and this factor was not associated with outcomes in adjusted analyses (Table 2).

Given the temporal nature of the COVID-19 pandemic in the US, we calculated division-level 30-day mortality rates stratified by calendar time (Figure 2C). Across the nation, adjusted 30-day mortality rates improved over the summer (aOR, 0.38; 95% CI, 0.28-0.51) and into the fall (aOR, 0.32; 95% CI, 0.17-0.58) compared with the spring. Post hoc analyses suggested that adjustment for this strong temporal trend was responsible for the lack of statistically significant differences between census divisions. These temporal improvements were similar across the 9 census divisions. However, 2 divisions (Mountain and West North Central, notably geographically next to each other) had the lowest unadjusted 30-day mortality rates in the spring and summer and then dramatically increased to having the highest mortality rates in the fall. Given this observation, we conducted a post hoc analysis comparing the adjusted odds of 30-day mortality between these 2 divisions combined vs all other divisions combined, stratified by time (eTable 4 in Supplement 1). Although the aOR increased from the summer (aOR, 0.65; 95% CI, 0.25-1.73) to the fall (aOR, 2.62; 95% CI, 0.86-8.02), the differences across time were not statistically significant (*P *for interaction = .13), likely because of relatively small numbers of patients reported from these 2 divisions (59 patients).

## Discussion

This is the first study from the CCC19 to describe spatiotemporal trends in COVID-19 outcomes among patients with cancer in the United States. While adjusting for patient-level, center-level, and US census division–level factors in multivariable models, several important findings emerged from our analyses. First, patient-level factors were approximately evenly distributed across all 4 US census regions with the exceptions of race and ethnicity, reflecting the underlying population in each region.

Second, the pattern of spread of the COVID-19 case rate seen across the US was also reflected in our registry and not associated with outcomes. In our cohort, most patients in the Northeast were diagnosed in the spring of 2020, while the largest proportion of patients in the South and the West were diagnosed in the fall of 2020. Similarly, we observed differences in the use of anti–COVID-19 therapies across the nation, likely reflecting the evolving nature of the treatment paradigm as more efficacy data emerged.^10,18,19^ Specifically, a higher proportion of patients received hydroxychloroquine in the Northeast (23.7%) compared with the West (2.9%). This stems from the fact that hydroxychloroquine was part of the treatment paradigm against COVID-19 early on, but its use waned as data on its nonbeneficial efficacy emerged.^10,19^ Remdesivir, however, was used proportionately more in the West (13.3%), which had higher rates of COVID-19 infection in the latter half of 2020, which corresponds with the US Food and Drug Administration approval in October 2020.

Third, while there was substantial heterogeneity in outcomes across cancer centers after adjustment for patient-level factors including calendar time, no association was observed between center-level factors and receipt of mechanical ventilation, ICU admission, or death. The exception was significantly lower odds of mechanical ventilation, ICU admission, and death in centers classified as RUCC 3 compared with centers classified as RUCC 1. This result is potentially explained by our post hoc analysis, which found that patients in RUCC 3–classified centers had a lower initial severity of COVID-19 compared with centers with RUCC 1 or 2. It is well known that social determinants of health, access to care, and many health outcomes vary across geographic regions.^20,21,22^ It is possible that the RUCC classification scheme is serving as a proxy for unmeasured patient and community factors (eg, RUCC 3 representing higher socioeconomic status in suburban areas), driving the differential outcomes in our cohort of patients with cancer. These potential unmeasured factors may also explain the substantial variation in outcomes observed across centers in unadjusted and adjusted models. It is noteworthy that the differences in outcomes in unadjusted models were attenuated in fully adjusted models with center-level and region-level covariates, suggesting that possible confounders include more patient-specific social and environmental aspects rather than specifics pertaining to their demographic characteristics or underlying cancer history and treatment.

Finally, although overall mortality rates improved over time as the pandemic continued, we did not observe substantial differences in the mortality rates among patients with cancer and COVID-19 residing in different census divisions when adjusting for time. This is notable, as different regions likely experienced resource challenges at different times of the year as COVID-19 cases swept the country; those resource limitations did not manifest in worse patient outcomes in our study. However, 2 divisions (Mountain and West North Central) had the lowest unadjusted 30-day mortality rates in the spring and summer and then markedly increased to having the highest mortality rates in the fall, although this trend did not differ significantly from other divisions, potentially because of small numbers and limited statistical power. A study from Colorado used a geodatabase of 35 environmental, socioeconomic, topographic, and demographic variables to explain the spatial variability of COVID-19 incidence and found that it intensified in mountain communities west of Denver and along the Urban Front Range,^23^ suggesting that hot spots within certain states may be driving the numbers.

### Strengths and Limitations

Notable strengths of our study include the detailed patient-level factors obtained on a large number of patients equally distributed across the United States. The multivariable mixed-effects model included cancer center–level variables and census division–level variables to embrace more community and social metrics in the context of where care was provided. This extends our previously published work beyond individual patient-level analyses.^2,3^ The analysis of heterogeneity in outcomes among the 83 cancer care centers warrants further detailed explorations and cooperation on the part of National Cancer Institute, Association of American Cancer Institutes, American Cancer Society, American Society of Clinical Oncology, and other leading clinical oncology organizations and leaders.

Our study has several limitations, including those associated with large registry cohorts: reliance on medical professionals and/or trainees for identifying and reporting all potential cases, reporting errors, and missing data. Given the voluntary nature of case reporting, the issue of generalizability to all patients with cancer is raised, and observations should be interpreted with caution. To mitigate potential bias in reporting errors, we implemented a quality scoring system of reports and excluded those with poor scores. Additionally, we used multiple imputation to account for missing data. Cancer center–level factors (RUCC and SVI) attempt to evaluate associations of socioeconomic factors with outcomes but likely do not reflect the full granularity of reports represented in our cohort. This is because cancer care centers (both community and academic) are often located in predominantly suburban and urban areas, and patients from rural areas travel into the hubs for care. Additionally, because of regulatory aspects and the Health Insurance Portability and Accountability Act protections of registry cohorts, the SVI and RUCC analysis was restricted to location of the cancer center, not geographic residence of the patient and related characteristics of their home community. A per-patient analysis of socioeconomic factors would provide more confidence in evaluating these components.

## Conclusions

In conclusion, we did not observe significant differences in rates of mechanical ventilation, ICU admission, or death among patients with cancer and COVID-19 across US census regions or divisions over time. Patients treated in centers located in less densely populated areas had significantly improved outcomes compared with patients treated in centers in more densely populated areas. Substantial heterogeneity in outcomes across all cancer care delivery centers was observed. These findings can inform future collaborative efforts across cancer care delivery centers to monitor outcomes on a per-center basis and collect site-specific information, such as number of positive cases, surrogates for resource strain, and change rate in cancer screening. Additionally, comparing guidelines and best practices for treatment of patients with cancer and COVID-19 and exploring socioeconomic and health determinants that may be unique to patients with cancer and COVID-19 may reveal mechanisms for the observed heterogeneity.

## Notes

Supplement 1. **eAppendix 1.** Alphabetical List of Participants by Institution That Contributed at Least 1 Record to the Analysis **eAppendix 2. **Statistical Analysis Plan **eMethods. **Details on Social Vulnerability Index and Rural-Urban Continuum Code **eTable 1.** Average Rate of SARS-CoV-2 Diagnosis in United States Census Divisions (Cases per Million Population), Stratified by Calendar Time **eTable 2.** Patients’ Severity of COVID-19 at Presentation by the Rural-Urban Continuum Code of the Center From Which Patients Were Reported **eTable 3.** Associations of Census Division With 30-Day All-Cause Mortality (Primary Outcome) and the Composite Outcome (Secondary), Adjusted for Patient-Level Characteristics Only **eTable 4.** Adjusted Odds Ratios Comparing the Odds of 30-Day All-Cause Mortality Between the West North Central and Mountain Census Divisions Combined vs All Other Census Divisions Combined, Stratified by Month of COVID-19 Diagnosis **eFigure.** Inclusion and Exclusion Criteria

Supplement 2.
Nonauthor Collaborators
