# Error in Nonauthor Collaborator Name

**DOI:** 10.1001/jamanetworkopen.2022.17965

**Published:** 2022-05-25

In the Original Investigation titled “Assessment of Regional Variability in COVID-19 Outcomes Among Patients With Cancer in the United States,”^1^ published January 4, 2022, there was an error in a nonauthor collaborator name in Supplement 2. Pier Vitale should have appeared as Pier Vitale Nuzzo. This article has been corrected.^1^
