# Supplementary material for: Assessment of Regional Variability in COVID-19 Outcomes Among Patients With Cancer in the United States
Source: JAMA Netw Open. 2022 Jan 4;5(1):e2142046. doi: 10.1001/jamanetworkopen.2021.42046 (PMC8728628; doi:10.1001/jamanetworkopen.2021.42046)
Supplement: Supplement 1. — eAppendix 1. Alphabetical List of Participants by Institution That Contributed at Least 1 Record to the Analysis eAppendix 2. Statistical Analysis Plan eMethods. Details on Social Vulnerability Index and Rural-Urban Continuum Code eTable 1. Average Rate of SARS-CoV-2 Diagnosis in United States Census Divisions (Cases per Million Population), Stratified by Calendar Time eTable 2. Patients’ Severity of COVID-19 at Presentation by the Rural-Urban Continuum Code of the Center From Which Patients Were Reported eTable 3. Associations of Census Division With 30-Day All-Cause Mortality (Primary Outcome) and the Composite Outcome (Secondary), Adjusted for Patient-Level Characteristics Only eTable 4. Adjusted Odds Ratios Comparing the Odds of 30-Day All-Cause Mortality Between the West North Central and Mountain Census Divisions Combined vs All Other Census Divisions Combined, Stratified by Month of COVID-19 Diagnosis eFigure. Inclusion and Exclusion Criteria [file jamanetwopen-e2142046-s001.pdf]

# Supplemental Online Content

Hawley JE, Sun T, Chism DD, et al; COVID-19 and Cancer Consortium (CCC19). Assessment of regional variability in COVID-19 outcomes among patients with cancer in the United States. *JAMA Netw Open.* 2022;5(1):e2142046. doi:10.1001/jamanetworkopen.2021.42046

**eAppendix 1.** Alphabetical List of Participants by Institution That Contributed at Least 1 Record to the Analysis

**eAppendix 2.** Statistical Analysis Plan

**eMethods.** Details on Social Vulnerability Index and Rural-Urban Continuum Code

**eTable 1.** Average Rate of SARS-CoV-2 Diagnosis in United States Census Divisions (Cases per Million Population), Stratified by Calendar Time

**eTable 2.** Patients' Severity of COVID-19 at Presentation by the Rural-Urban Continuum Code of the Center From Which Patients Were Reported

**eTable 3.** Associations of Census Division With 30-Day All-Cause Mortality (Primary Outcome) and the Composite Outcome (Secondary), Adjusted for Patient-Level Characteristics Only

**eTable 4.** Adjusted Odds Ratios Comparing the Odds of 30-Day All-Cause Mortality Between the West North Central and Mountain Census Divisions Combined vs All Other Census Divisions Combined, Stratified by Month of COVID-19 Diagnosis

**eFigure.** Inclusion and Exclusion Criteria

This supplemental material has been provided by the authors to give readers additional information about their work.

**eAppendix 1.** Alphabetical List of Participants by Institution That Contributed at Least 1 Record to the Analysis

**Bolded** = site PI/co-PIs; site co-investigators are listed alphabetically by last name.

1. **Balazs Halmos, MD; Amit Verma, MBBS**; Benjamin A. Gartrell, MD; Sanjay Goel, MBBS; Nitin Ohri, MD; R. Alejandro Sica, MD; Astha Thakkar, MD (Albert Einstein College of Medicine, Montefiore Medical Center, Bronx, NY, USA)
2. **Keith E. Stockerl-Goldstein, MD**; Omar Butt, MD, PhD; Jian L. Campian, MD, PhD; Mark A. Fiala, MSW; Jeffrey P. Henderson, MD, PhD; Ryan Monahan, MBA; Alice Y. Zhou, MD, PhD (Alvin J. Siteman Cancer Center at Washington University School of Medicine and Barnes-Jewish Hospital, St. Louis, MO, USA)
3. **Michael A. Thompson, MD, PhD, FASCO**; Pamela Bohachek, RN; Daniel Mundt, MD; Mitrianna Streckfuss, MPH; Eyob Tadesse, MD (Aurora Cancer Care, Advocate Aurora Health, Milwaukee, WI, USA)
4. **Philip E. Lammers, MD, MSCI** (Baptist Cancer Center, Memphis, TN, USA)
5. **Orestis A. Panagiotou, MD, PhD**; Pamela C. Egan, MD; Dimitrios Farmakiotis, MD, FACP, FIDSA; Hina Khan, MD; Adam J. Olszewski, MD (Brown University and Lifespan Cancer Institute, Providence, RI, USA)
6. **Arturo Loaiza-Bonilla, MD, MSEd, FACP** (Cancer Treatment Centers of America, AZ/GA/IL/OK/PA, USA)
7. **Salvatore A. Del Prete, MD**; Michael H. Bar, MD, FACP; Anthony P. Gulati, MD; K. M. Steve Lo, MD; Suzanne J. Rose, MS, PhD, CCRC, FACRP; Jamie Stratton, MD; Paul L. Weinstein, MD (Carl & Dorothy Bennett Cancer Center at Stamford Hospital, Stamford, CT, USA)
8. **Paolo Caimi, MD**; Jill S. Barnholtz-Sloan, PhD; Jorge A. Garcia, MD, FACP; John M. Nakayama, MD (Case Comprehensive Cancer Center at Case Western Reserve University/University Hospitals, Cleveland, OH, USA)
9. **Shilpa Gupta, MD**; Nathan A. Pennell, MD, PhD, FASCO; Manmeet S. Ahluwalia, MD, FACP; Scott J. Dawsey, MD; Christopher A. Lemmon, MD; Amanda Nizam, MD (Cleveland Clinic, Cleveland, OH, USA)
10. **Claire Hoppenot, MD; Ang Li, MD, MS** (Dan L Duncan Comprehensive Cancer Center at Baylor College of Medicine, Houston, TX, USA)
11. **Toni K. Choueiri, MD**; Ziad Bakouny, MD, MSc; Gabrielle Bouchard, BS; Fiona J. Busser, BA; Jean M. Connors, MD; Catherine R. Curran, BA; George D. Demetri, MD, FASCO; Antonio Giordano, MD, PhD; Kaitlin Kelleher, BA; Anju Nohria, MD; Andrew Schmidt, MD; Grace Shaw, BA; Eli Van Allen, MD; Pier Vitale Vincent Xu, MD; Rebecca L. Zon, MD (Dana-Farber Cancer Institute, Boston, MA, USA)
12. **Tian Zhang, MD, MHS**; Susan Halabi, PhD, FASCO (Duke Cancer Institute at Duke University Medical Center, Durham, NC, USA)
13. **John C. Leighton Jr, MD, FACP** (Einstein Healthcare Network, Philadelphia, PA, USA)
14. **Gary H. Lyman, MD, MPH, FASCO, FRCP**; Jerome J. Graber MD, MPH; Petros Grivas, MD, PhD; Ali Raza Khaki, MD; Elizabeth T. Loggers, MD, PhD; Ryan C. Lynch, MD; Elizabeth S. Nakasone, MD, PhD; Michael T. Schweizer, MD; Lisa Tachiki, MD; Shaveta Vinayak, MD,

MS; Michael J. Wagner, MD; Albert Yeh, MD (Fred Hutchinson Cancer Research Center/University of Washington/Seattle Cancer Care Alliance, Seattle, WA, USA)
15. **Na Tosha N. Gatson, MD, PhD, FAAN** (Geisinger Health System, PA, USA)
16. **Sharad Goyal, MD; Minh-Phuong Huynh-Le, MD, MAS** (George Washington University, Washington, DC, USA)
17. **Lori J. Rosenstein, MD** (Gundersen Health System, WI, USA)
18. **Peter Paul Yu, MD, FACP, FASCO**; Jessica M. Clement, MD; Ahmad Daher, MD; Mark Dailey, MD; Rawad Elias, MD; Asha Jayaraj, MD; Emily Hsu, MD; Alvaro G. Menendez, MD; Joerg Rathmann, MD; Oscar Serrano, MD (Hartford HealthCare Cancer Institute, Hartford, CT, USA)
19. **Clara Hwang, MD**; Shirish M. Gadgeel, MD; Sunny R K Singh, MD (Henry Ford Cancer Institute, Henry Ford Hospital, Detroit, MI, USA)
20. **Jessica E. Hawley, MD; Dawn Hershman, MD, MS, FASCO**; Melissa K. Accordino, MD, MS; Divaya Bhutani, MD; Gary K. Schwartz, MD (Herbert Irving Comprehensive Cancer Center at Columbia University, New York, NY, USA)
21. **Daniel Y. Reuben, MD, MS**; Mariam Alexander, MD, PhD; Sarah Mushtaq, MD (Hollings Cancer Center at the Medical University of South Carolina, Charleston, SC, USA)
22. **Eric H. Bernicker, MD** (Houston Methodist Cancer Center, Houston, TX, USA)
23. **John Deeken, MD**; Danielle Shafer, DO (Inova Schar Cancer Institute, Fairfax, VA, USA)
24. **Mark A. Lewis, MD; Terence D. Rhodes, MD, PhD**; David M. Gill, MD; Clarke A. Low, MD (Intermountain Health Care, Salt Lake City, UT, USA)
25. **Sandeep H. Mashru, MD**; Abdul-Hai Mansoor, MD (Kaiser Permanente Northwest, OR/WA, USA)
26. **Howard A. Zaren, MD, FACS**; Stephanie J. Smith, RN, MSN, OCN (Lewis Cancer & Research Pavilion @ St. Joseph's/Candler, Savannah, GA, USA)
27. **Gayathri Nagaraj, MD**; Mojtaba Akhtari, MD; Eric Lau, DO; Mark E. Reeves, MD, PhD (Loma Linda University Cancer Center, Loma Linda, CA, USA)
28. **Stephanie Berg, DO**; Destry Elms, MD (Loyola University Medical Center, Maywood, IL, USA)
29. **Alicia K. Morgans, MD, MPH; Firas H. Wehbe, MD, PhD**; Jessica Altman, MD; Michael Gurley, BA; Mary F. Mulcahy, MD (Lurie Cancer Center at Northwestern University, Chicago, IL, USA)
30. **Eric B. Durbin, DrPH, MS** (Markey Cancer Center at the University of Kentucky, Lexington, KY, USA)
31. **Amit A. Kulkarni, MD**; Heather H. Nelson, PhD, MPH; Zohar Sachs, MD, PhD; Surbhi Shah, MD (Masonic Cancer Center at the University of Minnesota, Minneapolis, MN, USA)
32. **Rachel P. Rosovsky, MD, MPH; Kerry Reynolds, MD**; Aditya Bardia, MD; Genevieve Boland, MD, PhD, FACS; Justin Gainor, MD; Leyre Zubiri, MD, PhD (Massachusetts General Hospital Cancer Center, Boston, MA, USA)
33. **Thorvardur R. Halfdanarson, MD**; Tanios Bekaii-Saab, MD; Aakash Desai, MD, MPH; Zhuoer Xie, MD, MS (Mayo Clinic, AZ/FL/MN, USA)
34. **Ruben A. Mesa, MD, FACP**; Mark Bonnen, MD; Daruka Mahadevan, MD, PhD; Amelie G. Ramirez, DrPH, MPH; Mary Salazar, ANP; Dimpy P. Shah, MD, PhD; Pankil K. Shah, MD,

MSPH (Mays Cancer Center at UT Health San Antonio MD Anderson Cancer Center, San Antonio, TX, USA)

35. **Bryan Faller, MD** (Missouri Baptist Medical Center, St. Louis, MO, USA)
36. **Rana R. McKay, MD**; Archana Ajmera, MSN, ANP-BC, AOCNP; Sharon S. Brouha, MD, MPH; Angelo Cabal, BS; Albert Hsiao, MD, PhD; Seth Kligerman, MD; Justin A. Shaya, MD (Moores Comprehensive Cancer Center at the University of California, San Diego, La Jolla, CA, USA)
37. **Lisa B. Weissmann, MD**; Chinmay Jani, MD; Carey C. Thomson, MD, FCCP, MPH (Mount Auburn Hospital, Cambridge, MA, USA)
38. **Jeanna Knoble, MD**; Mary Grace Glace, RN; Cameron Rink, PhD, MBA; Karen Stauffer, RN; Rosemary Zacks, RN (Mount Carmel Health System, Columbus, OH, USA)
39. **Sibel Blau, MD** (Northwest Medical Specialties, Tacoma, WA, USA)
40. **Monika Joshi, MD, MRCP**; Harry Menon, DO, MPH; Marc A. Rovito, MD, FACP (Penn State Health/Penn State Cancer Institute/St. Joseph Cancer Center, PA, USA)
41. **Elizabeth A. Griffiths, MD**; Amro Elshoury, MBBCh (Roswell Park Comprehensive Cancer Center, Buffalo, NY, USA)
42. **Salma K. Jabbour, MD**; Christian F. Misdary, MD; Mansi R. Shah, MD (Rutgers Cancer Institute of New Jersey at Rutgers Biomedical and Health Sciences, New Brunswick, NJ, USA)
43. **Babar Bashir, MD, MS**; Christopher McNair, PhD; Sana Z. Mahmood, BA, BS; Vasil Mico, BS; Andrea Verghese Rivera, MD (Sidney Kimmel Cancer Center at Thomas Jefferson University, Philadelphia, PA, USA)
44. **Daniel B. Flora, MD, PharmD**; Goetz Kloecker, MD; Barbara B. Logan, MS; Chaitanya Mandapakala, MD (St. Elizabeth Healthcare, Edgewood, KY, USA)
45. **Sumit A. Shah, MD, MPH**; Elwyn C. Cabebe, MD; Michael J. Glover, MD; Alokkumar Jha, PhD; Lidia Schapira, MD, FASCO; Julie Tsu-Yu Wu, MD, PhD (Stanford Cancer Institute at Stanford University, Palo Alto, CA, USA)
46. **Suki Subbiah, MD** (Stanley S. Scott Cancer Center at LSU Health Sciences Center, New Orleans, LA, USA)
47. **Gilberto de Lima Lopes Jr., MD, MBA, FAMS, FASCO** (Sylvester Comprehensive Cancer Center at the University of Miami Miller School of Medicine, Miami, FL, USA)
48. **Sanjay G. Revankar, MD, FIDSA** (The Barbara Ann Karmanos Cancer Institute at Wayne State University School of Medicine, Detroit, MI, USA)
49. **Daniel G. Stover, MD**; Daniel Addison, MD; James L. Chen, MD; Margaret E. Gatti-Mays, MD; Sachin R. Jhawar, MD; Vidhya Karivedu, MBBS; Maryam B. Lustberg, MD, MPH; Joshua D. Palmer, MD; Sarah Wall, MD; Nicole Williams, MD (The Ohio State University Comprehensive Cancer Center, Columbus, OH, USA)
50. **Elizabeth Wulff-Burchfield, MD**; Anup Kasi MD, MPH (The University of Kansas Cancer Center, Kansas City, KS, USA)
51. **Natasha Edwin, MD**; Melissa Smits, APC (ThedaCare Cancer Care, Appleton, WI, USA)
52. **David D. Chism, MD**; Susie Owenby, RN, CCRP (Thompson Cancer Survival Center, Knoxville, TN, USA)

53. **Deborah B. Doroshow, MD, PhD**; Matthew D. Galsky, MD; Michael Wotman, MD; Huili Zhu, MD (Tisch Cancer Institute at the Icahn School of Medicine at Mount Sinai, New York, NY, USA)
54. **Julie C. Fu, MD**; Alyson Fazio, APRN-BC; Kathryn E. Huber, MD; Mark H. Sueyoshi, MD (Tufts Medical Center Cancer Center, Boston and Stoneham, MA, USA)
55. **Jonathan Riess, MD, MS**; Kanishka G. Patel, MD (UC Davis Comprehensive Cancer Center at the University of California at Davis, CA, USA)
56. **Samuel M. Rubinstein, MD**; William A. Wood, MD, MPH; Christopher Jensen, MD; Vaibhav Kumar, MD (UNC Lineberger Comprehensive Cancer Center, Chapel Hill, NC, USA)
57. **Trisha M. Wise-Draper, MD, PhD**; Syed A. Ahmad, MD, FACS; Punita Grover, MD; Shuchi Gulati, MD; Jordan Kharofa, MD; Tahir Latif, MBBS, MBA; Michelle Marcum, MS; Cathleen Park, MD, Hira G. Shaikh; MD (University of Cincinnati Cancer Center, Cincinnati, OH, USA)
58. **Daniel W. Bowles, MD**; Christoper L. Geiger, MD (University of Colorado Cancer Center, Aurora, CO, USA)
59. **Merry-Jennifer Markham, MD, FACP, FASCO**; Rohit Bishnoi, MD; Atlantis D. Russ, MD, PhD; Chintan Shah, MD (University of Florida Health Cancer Center, Gainesville, FL, USA)
60. **Jared D. Acoba, MD**; Young Soo Rho, MD, CM (University of Hawai'i Cancer Center, Honolulu, HI, USA)
61. **Lawrence E. Feldman, MD; Kent F. Hoskins, MD**; Gerald Gantt Jr., MD; Li C. Liu, PhD; Mahir Khan, MD; Ryan H. Nguyen, DO; Mary Pasquinelli, APN, DNP; Candice Schwartz, MD; Neeta K. Venepalli, MD, MBA (University of Illinois Hospital & Health Sciences System, Chicago, IL, USA)
62. **Praveen Vikas, MD** (University of Iowa Holden Comprehensive Cancer Center, Iowa City, IA, USA)
63. **Christopher R. Friese, PhD, RN, AOCN, FAAN; Leslie A. Fecher, MD** (University of Michigan Rogel Cancer Center, Ann Arbor, MI, USA)
64. **Blanche H. Mavromatis, MD**; Ragneel R. Bijjula, MD; Qamar U. Zaman, MD (UPMC Western Maryland, Cumberland, MD, USA)
65. **Jeremy L. Warner, MD, MS, FAMIA, FASCO**; Alex Cheng, PhD; Elizabeth J. Davis, MD; Stephany N. Duda, PhD, MS; Kyle T. Enriquez, MSc BS; Benjamin French, PhD; Erin A. Gillaspie, MD, MPH; Cassandra Hennessy, MS; Daniel Hausrath, MD; Chih-Yuan Hsu, PhD; Douglas B. Johnson, MD, MSCI; Xuanyi Li, BA; Sanjay Mishra, MS, PhD; Sonya A. Reid, MD, MPH; Brian I. Rini, MD, FACP, FASCO; David A. Slosky, MD; Yu Shyr, PhD; Carmen C. Solorzano, MD, FACS; Tianyi Sun, MS; Matthew D. Tucker, MD; Karen Vega-Luna, MA; Lucy L. Wang, BA (Vanderbilt-Ingram Cancer Center at Vanderbilt University Medical Center, Nashville, TN, USA)
66. **Hagen F. Kennecke, MD, MHA, FRCPC**; David M. Aboulafia, MD; Brett A. Schroeder, MD (Virginia Mason Cancer Institute, Seattle, WA, USA)
67. **Matthew Puc, MD**; Theresa M. Carducci, MSN, RN, CCRP; Karen J. Goldsmith, BSN, RN; Susan Van Loon, RN, CTR, CCRP (Virtua Health, Marlton, NJ, USA)
68. **Umit Topaloglu, PhD, FAMIA**; Saif I. Alimohamed, MD (Wake Forest Baptist Comprehensive Cancer Center, Winston-Salem, NC, USA)

© 2022 Hawley JE et al. *JAMA Network Open.*

69. **Robert L. Rice, MD, PhD** (WellSpan Health, York, PA, USA)
70. **Wilhelmina D. Cabalona, MD**; Christine Pilar, BS, CCRC, ACRP-PM (Wentworth-Douglass Hospital, Dover, NH, USA)
71. **Prakash Peddi, MD; Lane R. Rosen, MD**; Briana Barrow McCollough, BSc, CCRC (Willis-Knighton Cancer Center, Shreveport, LA, USA)
72. **Mehmet A. Bilen, MD**; Deepak Ravindranathan, MD, MS (Winship Cancer Institute of Emory University, Atlanta, GA, USA)
73. **Navid Hafez, MD, MPH**; Roy Herbst, MD, PhD; Patricia LoRusso, DO, PhD; Tyler Masters, MS; Catherine Stratton, BA (Yale Cancer Center at Yale University School of Medicine, New Haven, CT, USA)

**eAppendix 2.** Statistical Analysis Plan

| | |
|---|---|
| Approved Project Title | Regional Variability and Urban-Rural Health Disparities in Outcomes of Patients with Cancer and COVID-19 |
| Approved Project PIs | Jessica Hawley and Clara Hwang |
| 1 (a) Manuscript title | Assessment of US Regional Variability in COVID-19 Outcomes Among Patients With Cancer |
| 3 Objectives<br>State specific objectives, including any prespecified hypotheses | H0: Clinical outcomes and US census region are independent in patients with cancer and COVID-19 in adjusted models (no difference in death (30-day all-cause mortality) or composite endpoint (death, rate of ICU admission, rate of mechanical ventilation).<br>HA: Clinical outcomes and US census region are associated in patients with cancer and COVID-19. |
| 4 Study design | Retrospective, multi-center cohort study |
| 5 Setting | Cohort study of patients with current or past history of cancer and lab-confirmed SARS-CoV-2 infection from >120 participating CCC19 sites. Data analyzed from March 15, 2020 – November 30, 2020. Registry built and maintained as electronic REDCap database at VUMC. |
| 6 Participants | |
| (a) Give the eligibility criteria, and the sources and methods of selection of participants. Describe methods of follow-up | Subjects included in this analysis were those with laboratory-confirmed SARS-CoV-2 infection and past or current history of invasive malignancy, from the U.S., and entered into the CCC19 database between 3/17/2020 and 11/30/2020 with follow-up data reported through 12/31/2020. We excluded records of presumptive COVID-19 cases, patients < 18 years of age, patients with incomplete follow-up data, cases of non-invasive cancers and premalignant conditions or non-melanoma non-invasive skin cancers, cases from outside the U.S., and records with quality score >4. |
| (b) For matched studies, give matching criteria and number of exposed and unexposed | Not a matched study. |
| 7 Variables | |
| (a) Outcomes | Primary outcomes: 30-day all-cause mortality (binary)<br>Secondary outcomes: composite binary outcome (death at any time, ICU admission, or mechanical ventilation), and individual components of composite outcome to assess overall medical burden by U.S. Census subregion.<br>Analysis of outcomes conducted overall (all time periods) and by three prespecified time periods. |
| (b) Exposures | 9 US Census Subregions (Defined as per list below) |
| (c) Potential confounders | **Census Region Level:** |

© 2022 Hawley JE et al. JAMA Network Open.

| | |
|---|---|
| | • Average daily new COVID-19 case rate per 1,000,000 people (continuous)<br>• Population density for region (continuous)<br><br>**Center-level:**<br>• SVI (continuous)<br>• RUCC code (ordinal)<br>• Type of Center – academic vs. community (binary)<br><br>**Individual-level:**<br>• Age<br>• sex<br>• race/ethnicity<br>• smoking<br>• obesity<br>• specific comorbidities (CV, pulmonary, renal, DM)<br>• type of malignancy (solid vs. heme)<br>• cancer status<br>• ECOG status<br>• type of cancer-directed therapy or surgery<br>• COVID-19 treatments<br><br>**Time intervals** (use every 3 months = 3 time periods)<br>• March, April, May<br>• June, July, August<br>• Sept, Oct, Nov |
| (d) Effect modifiers | None |
| (e) Diagnostic criteria (if applicable) | Laboratory-confirmed positive for SARS-CoV-2 |
| 8 Data sources/measurement For each variable of interest, give sources of data and details of methods of assessment (measurement). Describe comparability of assessment methods if there is more than one group | Variables of interest are defined by the CCC19 database registry and entered by each local site's data abstractor.<br>-race/ethnicity self-reported (5 categories)<br>-smoking (never v. ever v. missing)<br>-obesity (y/n/missing)<br>-specific comorbidities (CV, pulm, renal, DM, missing)<br>-cancer status (active and responding, active and stable, active and progressing vs. remission/NED, unknown, missing)<br>-ECOG (0, 1, ≥ 2, unknown, missing)<br>-type of malignancy (solid vs. heme)<br>-modality of active anti-cancer therapy (categorical: none, cytotoxic chemo, immunotherapy, targeted, endocrine, locoregional, other, missing/unknown) |

© 2022 Hawley JE et al. JAMA Network Open.

| | |
|---|---|
| | -COVID-19 tx (categorical: use JCO paper groupings)<br>-Rural-urban status (center-level: ordinal – 1, 2, 3)<br>-Neighborhood (cont: use center-level SVI)<br>-Population density by US census subregion (continuous)<br>-New COVID19 ave daily case over 3 time intervals by region (continuous) |
| 9 Bias<br>Describe any efforts to address potential sources of bias | Adjustment for covariates in multivariable models. |
| 10 Study size<br>Explain how the study size was arrived at | Case volume dependent on data abstracters at each site. |
| 11 Quantitative variables<br>Explain how quantitative variables will be handled in the analyses. If applicable, describe which groupings will be chosen and why | As per above |
| 12 Statistical methods | |
| (a) Describe all statistical methods, including those to be used to control for confounding | Covariates (listed above under potential confounders) and binary outcomes will be summarized across 9 census subregions using standard descriptive statistics. Multivariable generalized linear mixed-effects models (with a logit link for binary outcomes and center-level random effects) will be used to (1) estimate adjusted covariate-outcome associations and (2) estimate adjusted subregion-level outcome rates overall and at 3-month time intervals. For secondary outcomes, the model will include an offset for (log) follow-up time. A list of potential tables and figures is provided below. |
| (b) Describe any methods that will be used to examine subgroups and interactions | None |
| (c) Explain how missing data will be addressed | Multiple imputation will be used to impute missing and unknown data for all variables included in the analysis, with some exceptions: unknown ECOG performance score and unknown cancer status will not be imputed and treated as a separate category in analyses. Imputation will be performed on the largest dataset possible (that is, after removing test cases and other manual exclusions, but before applying specific exclusion criteria). At least 10 imputations will be generated. |

© 2022 Hawley JE et al. JAMA Network Open.

| | |
|---|---|
| (d) If applicable, explain how loss to follow-up will be addressed | Excluded if no data on 30-day follow-up form. We will extend follow-up time (through 12/31/2020) to ensure that follow-up for the primary outcome, 30-day mortality, is complete to the best of our ability. |
| (e) Describe any sensitivity analyses | None |
| Date approved | January 22, 2021 |

**US Census Regions / Division:**

- Region I: Northeast
  - New England: Connecticut, Maine, Massachusetts, New Hampshire, Rhode Island, Vermont
  - Middle Atlantic: New Jersey, New York, Pennsylvania
- Region II: Midwest
  - East North Central: Indiana, Illinois, Michigan, Ohio, Wisconsin
  - West North Central: Iowa, Kansas, Minnesota, Missouri, Nebraska, North Dakota, South Dakota
- Region III: South
  - South Atlantic: Delaware, DC, Florida, Georgia, Maryland, North Carolina, South Carolina, Virginia, West Virginia
  - East South Central: Alabama, Kentucky, Mississippi, Tennessee
  - West South Central: Arkansas, Louisiana, Oklahoma, Texas
- Region IV: West
  - Mountain: Arizona, Colorado, Idaho, New Mexico, Montana, Utah, Nevada, Wyoming
  - Pacific: Alaska, California, Hawaii, Oregon, Washington

© 2022 Hawley JE et al. JAMA Network Open.

The **COVID-19** & **Cancer** Consortium

# Approved Project Variables

Once the project design and SAP have been approved for your project, this document will be used to specify the exact outcomes and variables to be used in your analysis. Please provide as much information as you can, including the existing variable name if you know it. Use of existing variables will decrease the amount of time that it takes to get your project to the analysis phase, but we will endeavor to add any needed derived variables based on your project needs. Existing variables can be found in two places within the GitHub repo: the **Data Dictionary** (“`CCC19_DataDictionary.csv`”), which includes the native variables found in the survey, and the list of **Derived Variables** (“`CCC19_Derived_Variables_Spreadsheet.xlsx`”).

***Please fill out and return this form to Jeremy Warner (jeremy.warner@vumc.org), Sanjay Mishra (sanjay.mishra.1@vumc.org) and your assigned biostatistics lead.***

*If you need extra rows just hit Tab when your cursor is in the bottom right cell.*

| Outcome description | Outcome variable name | Outcome values | Additional Details |
|---|---|---|---|
| **Derived variable indicating whether patient has died within 30 days of COVID-19 diagnosis (default = No)** | der_dead30 | 0 = No; 1 = Yes; 99 = Unknown | |
| **Derived dead/alive variable** | *der_deadbinary* | 0 = No; 1 = Yes; 99 = Unknown | |
| **Derived variable indicating whether patients required mechanical ventilation** | der_mv | 0 = No; 1 = Yes; 99 = Unknown | |
| **Derived variable indicating time in ICU** | der_ICU | 0 = No; 1 = Yes; 99 = Unknown | |
| **Derived variable of composite outcome** | der_composite_ICU_mv_death (der_composite.3.v2 is the variable name in R script) | 0 = No; 1 = Yes; 99 = Unknown | Derived with: *der_deadbinary, der_mv, der_ICU* |
| **Median F/U in days** | der_days_fu *(der_median_fu is the variable name in R script)* | | |

| Covariate description | Variable name | Covariate values | Additional Details |
|---|---|---|---|
| ***Patient-level covariates*** | | | |

© 2022 Hawley JE et al. JAMA Network Open.

| | | | |
|---|---|---|---|
| **Region of patient residence with ex-US collapsed** | der_region_v2 | Non-US; Other; Undesignated US; US Midwest; US Northeast; US South; US West | |
| **Region of patient residence with US and ex-US collapsed** | der_region_v3 | Non-US; Other; US | |
| **US Census Division** | der_division | East North Central; East South Central; Middle Atlantic; Mountain; New England; Pacific; South Atlantic; West North Central; West South Central | |
| **Age with imputation for categoricals** | *der_age_trunc (der_age is the variable name in the R script)* | Years (continuous 18-89; patients noted to be greater than 89 are set to be age = 90) | |
| ***Sex* (Recode other/prefer not to say gender --> missing*)*** | *der_sex* | *Male, Female* | |
| **Derived variable for race/ethnicity** | der_race | Non-Hispanic White; Hispanic; Non-Hispanic Black; Other | |
| **Derived variable for smoking status collapsing the current/former smoker variables** | der_smoking2 | Never; Current or Former; Unknown | |
| **Binary obesity (BMI >= 30 or checkbox checked) indicator** | der_obesity | 0 = No; 1 = Yes; 99 = Unknown | |
| **Cardiovascular comorbidity (CAD, CHF, Afib, arrhythmia NOS, PVD, CVA, cardiac disease NOS)** | der_card | 0 = No; 1 = Yes; 99 = Unknown | |
| **Derived variable indicating whether patient has pulmonary comorbidities** | der_pulm | 0 = No; 1 = Yes; 99 = Unknown | |
| **Renal comorbidities** | der_renal | 0 = No; 1 = Yes; 99 = Unknown | |
| **Derived variable indicating whether patient has diabetes mellitus** | der_dm2 | 0 = No; 1 = Yes; 99 = Unknown | |

© 2022 Hawley JE et al. JAMA Network Open.

| | | | |
|---|---|---|---|
| **Solid tumor indicator** | der_solid | 0 = No; 1 = Yes; | |
| **Hematologic malignancy indicator** | der_heme | 0 = No; 1 = Yes | |
| **Derived variable indicating cancer status** | der_cancer_status | 0 - Remission/NED; 1 - Active, stable/responding; 2 - Active, progressing; 99 - Unknown | |
| **Timing of cancer treatment relative to COVID-19, collapsed** | der_cancer_tx_timing_v3 | 0 = more than 3 months; 1 = 0-3 months; 88 = never or after COVID-19 diagnosis; 99 = unknown | |
| **Performance Status** | der_ecogcat2 | ECOG 0, 1, or 2+ | |
| **No cancer treatment in the 3 months prior to COVID-19** | der_cancertr_none | 0=No; 1=Yes; 99=Unknown | Derived with the following covariates:<br><br>der_any_cyto,<br>der_any_targeted,<br>der_any_endo,<br>der_any_immuno,<br>der_any_local,<br>der_any_other<br><br>Coded as 1 if all these variables are 0; coded as 0 if any of these variables is 1; coded as 99 if any of these variables is 99; otherwise, NA |
| **Any cytotoxic cancer treatment in the 3 months prior to COVID-19** | der_any_cyto | 0 = No; 1 = Yes; 99 = Unknown | |
| **Any targeted therapy in the 3 months prior to COVID-19** | der_any_targeted | 0 = No; 1 = Yes; 99 = Unknown | |
| **Any endocrine therapy in the 3 months prior to COVID-19** | der_any_endo | 0 = No; 1 = Yes; 99 = Unknown | |
| **Any immunotherapy in the 3 months prior to COVID-19** | der_any_immuno | 0 = No; 1 = Yes; 99 = Unknown | |
| **Any local therapy (surgery or RT) within 3 months** | der_any_local | 0 = No; 1 = Yes; 99 = Unknown | |
| **Any other cancer therapy in the 3 months prior to COVID-19** | der_any_other | 0 = No; 1 = Yes; 99 = Unknown | |
| **No COVID-19 treatment ever** | der_covidtr_none | 0 = No; 1 = Yes; 99 = Unknown | Derived with the following covariates:<br><br>der_rem,<br>der_hcq,<br>der_steroids_c19, |

© 2022 Hawley JE et al. JAMA Network Open.

| | | | |
|---|---|---|---|
| | | | der_other_tx_c19_v2<br><br>Coded as 1 if all these variables are 0; coded as 0 if any of these variables is 1; coded as 99 if any of these variables is 99; otherwise, NA |
| **Remdesivir as treatment for COVID-19 ever** | der_rem | 0 = No; 1 = Yes; 99 = Unknown | |
| **Hydroxychloroquine as COVID-19 treatment ever** | der_hcq | 0 = No; 1 = Yes; 99 = Unknown | |
| **Steroids as COVID-19 treatment ever** | der_steroids_c19 | 0 = No; 1 = Yes; 99 = Unknown | |
| **COVID-19 treatments other than HCQ, steroids, remdesivir** | der_other_tx_c19_v2 | 0 = No; 1 = Yes; 99 = Unknown | |
| **Trimester and year of diagnosis, using the most recent side of the interval as anchor** | der_tri_rt_dx | T1 2020; T2 2020; T3 2020; T1 2021 | |
| **Derived variable indicating cancer type** | der_ttype | Solid, Heme, Multiple | Derived with:<br><br>der_solid,<br>der_heme,<br>cancer_type_2<br><br>Coded as Solid if der_solid is 1 and cancer_type_2=" "; coded as Heme if der_heme is 1 and cancer_type_2=" "<br>Otherwise, coded as Multiple |
| **No cancer therapy, ever** | cancer_tx_never | 0 = No; 1 = Yes; | Derived with der_cancer_tx_timing_v3<br><br>No recent cancer therapy; directly mapped from cancer_tx_timing_v3 = 88 |
| **participating institution ID** | ccc19_institution[1] | | |
| **Derived hospitalized/not hospitalized variable** | der_hosp | 0 = No; 1 = Yes; 99 = Unknown | Used for imputation only |
| ***Center-level covariates*** | | | |
| **Social Vulnerability Index** | svi | Continuous | |
| **Rural-Urban Continuum Codes** | rucc | 1, Metro – 1 million population or more; 2, Metro – 250,000 to 1 million population; 3, Metro – fewer than 250,000 population | |
| **Type of center** | hosp_type | Academic; Community | |
| **Number of patients at each center** | volume | Integer (Patients) | Count the number of patients at each center within final dataset (with selected cohort), then link it to center-level data (using ccc19_institution from ccc19x and Center_ID from CCC19_center_level_covariates.v3_3.4.2021.xlsx<br><br>) |

© 2022 Hawley JE et al. JAMA Network Open.

| | | | |
|---|---|---|---|
| **Derived variable using state** | region | US Northeast; US Midwest; US South; US West | Derived with State |
| ***Census-level covariates*** | | | |
| **Average rate of SARS-CoV-2 diagnosis, per 100 cases per million population** | reg_daily_avg_newCOVID_v2 | Continuous | Derived with reg_T1_daily_avg_newCOVID_per_1mil, reg_T2_daily_avg_newCOVID_per_1mil, reg_T3_daily_avg_newCOVID_per_1mil der_tri_rt_dx<br><br>Derived after der_tri_rt_dx imputed |

[1]Raw variable

Once this form has been finalized, it will be signed by:

Approved:________________________________ Date: 11/17/2021

Core Lead, Informatics

Approved:________________________________ Date: 11/17/2021

Biostatistics Analyst

© 2022 Hawley JE et al. JAMA Network Open.

**eMethods.** Details on Social Vulnerability Index and Rural-Urban Continuum Code

The Centers for Disease Control and Prevention's Social Vulnerability Index (SVI) indicates the relative vulnerability of every U.S. Census tract (or county) according to ranks on 15 social factors in four domains: socioeconomic status, household composition and disability, minority status and language, and housing and transportation. An overall percentile rank (0-1) for the county where each center is located was used in this analysis. A SVI ranking of 0.85 indicates that 85% of tracts (or counties) in the state or nation are less vulnerable than the tract of interest and that 15% of tracts (or counties) in the state or nation are more vulnerable.

The U.S. Department of Agriculture's Economic Research Service's 2013 Rural-Urban Continuum Code (RUCC) classifies all counties in the U.S. by their official metro-nonmetro status, as defined by the Office of Management and Budget, with further breakdown by population resulting in nine RUCC codes (1-9). In this coding scheme, 1 represents counties in metropolitan areas with populations of 1 million, and 9 represents counties that are completely rural with populations of <2500, not adjacent to a metro area.

© 2022 Hawley JE et al. *JAMA Network Open.*

**eTable 1.** Average Rate of SARS-CoV-2 Diagnosis in United States Census Divisions (Cases per Million Population), Stratified by Calendar Time

| | Mar–May, 2020 | Jun–Aug, 2020 | Sep–Nov, 2020 |
|---|---|---|---|
| New England | 88 | 30 | 116 |
| Middle Atlantic | 182 | 134 | 302 |
| East North Central | 65 | 87 | 385 |
| West North Central | 33 | 81 | 351 |
| South Atlantic | 24 | 64 | 90 |
| East South Central | 40 | 198 | 312 |
| West South Central | 33 | 207 | 229 |
| Mountain | 13 | 58 | 169 |
| Pacific | 29 | 140 | 140 |

© 2022 Hawley JE et al. *JAMA Network Open.*

**eTable 2.** Patients' Severity of COVID-19 at Presentation by the Rural-Urban Continuum Code of the Center From Which Patients Were Reported

| | RUCC 1[a] | RUCC 2[b] | RUCC 3[c] |
|---|---|---|---|
| | n = 3820 | n = 666 | n = 263 |
| COVID-19 severity at presentation, n (%) | | | |
| Mild – no hospitalization indicated[d] | 1965 (51.4) | 330 (49.5) | 208 (79.1) |
| Moderate – hospitalization indicated[d] | 1407 (36.8) | 254 (38.1) | 47 (17.9) |
| Severe – ICU admission indicated[d] | 443 (11.6) | 80 (12.0) | 7 (2.7) |
| Missing/unknown, n (%) | 5 (0.1) | 2 (0.3) | 1 (0.4) |

ICU, intensive care unit; RUCC, rural-urban continuum code.

[a] Metro – 1 million population or more.

[b] Metro – 250,000 to 1 million population.

[c] Metro – fewer than 250,000 population.

[d] Whether or not it occurred.

© 2022 Hawley JE et al. *JAMA Network Open.*

**eTable 3.** Associations of Census Division With 30-Day All-Cause Mortality (Primary Outcome) and the Composite Outcome (Secondary), Adjusted for Patient-Level Characteristics Only

| | 30-day mortality | Composite outcome[a] |
|---|---|---|
| | OR[b] (95% CI) | OR[c] (95% CI) |
| Census division[d] | | |
| New England | 1.55 (0.89-2.70) | 1.56 (0.74-3.28) |
| Middle Atlantic | 1.64 (0.97-2.77) | 1.32 (0.67-2.62) |
| East North Central | Reference | Reference |
| West North Central | 1.16 (0.57-2.39) | 1.31 (0.58-2.92) |
| South Atlantic | 1.31 (0.73-2.35) | 1.41 (0.73-2.73) |
| East South Central | 1.58 (0.79-3.17) | 1.56 (0.68-3.58) |
| West South Central | 1.99 (0.94-4.23) | 1.89 (0.72-4.92) |
| Mountain | 1.10 (0.43-2.80) | 1.35 (0.48-3.82) |
| Pacific | 0.87 (0.43-1.76) | 0.76 (0.33-1.76) |

CI, confidence interval; OR, odds ratio.

[a] The composite outcome reflected the occurrence of any of the following: admission to an intensive care unit, receipt of mechanical ventilation, and total all-cause mortality. Analyses of the composite outcome were limited to 4,561 patients within non-missing data.

[b] Odds ratios greater than 1 indicate higher odds of 30-day all-cause mortality.

[c] Odds ratios greater than 1 indicate higher odds of admission to an intensive care unit, receipt of mechanical ventilation, or total all-cause mortality.

[d] Adjusted for age, sex, race and ethnicity, smoking status, obesity, cardiovascular comorbidities, pulmonary comorbidities, renal disease, diabetes mellitus, type of malignancy, cancer status, Eastern Cooperative Oncology Group performance status, anti-COVID-19 treatments, and month of COVID-19 diagnosis. P values for evaluating the null hypothesis of equality in odds ratios across census divisions (8 degrees of freedom): 30-day mortality, 0.42; composite outcome, 0.73.

© 2022 Hawley JE et al. *JAMA Network Open.*

**eTable 4.** Adjusted Odds Ratios Comparing the Odds of 30-Day All-Cause Mortality Between the West North Central and Mountain Census Divisions Combined vs All Other Census Divisions Combined, Stratified by Month of COVID-19 Diagnosis

| | OR[a] (95% CI) |
|---|---|
| March–May, 2020 | 1.12 (0.55-2.27) |
| June–August, 2020 | 0.65 (0.25-1.73) |
| September–November, 2020 | 2.62 (0.86-8.01) |

CI, confidence interval, OR, odds ratio.

[a] P value for evaluating the null hypothesis of equality in odds ratios across month of COVID-19 diagnosis: 0.13 (2 degrees of freedom).

© 2022 Hawley JE et al. *JAMA Network Open.*

**eFigure.** Inclusion and Exclusion Criteria

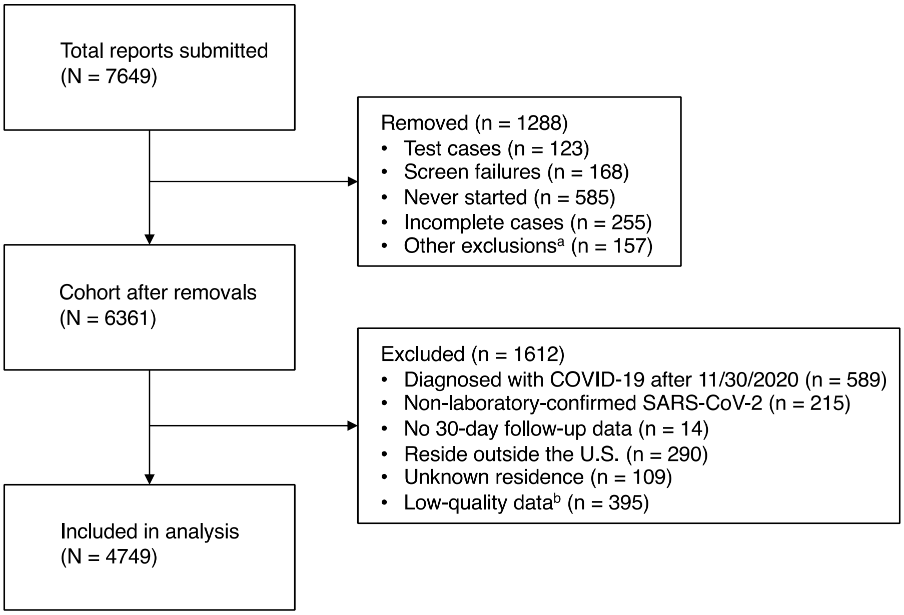


[a] Includes duplicate records, in situ solid malignancy, non-invasive non-melanoma skin cancer, precursor hematologic condition, benign hematologic condition, false-positive SARS-CoV-2 test, and non-CCC19 site.

[b] Quality score ≥5. Exclusions for low-quality data by census region: Northeast, 170/1734 (9.8%); Midwest, 101/1739 (5.8%); South, 80/974 (8.2%); West, 44/697 (6.3%).

© 2022 Hawley JE et al. *JAMA Network Open.*
