# Supplementary material for: Assessment of Regional Variability in COVID-19 Outcomes Among Patients With Cancer in the United States
Source: JAMA Netw Open. 2022 Jan 4;5(1):e2142046. doi: 10.1001/jamanetworkopen.2021.42046 (PMC8728628; doi:10.1001/jamanetworkopen.2021.42046)
Supplement: Supplement 2. — Nonauthor Collaborators [file jamanetwopen-e2142046-s002.pdf]

Supplemental Online Content: Nonauthor Collaborators

*Indicates required information. Only first name, last name, and suffix will appear in PubMed.

*Group Name(s): COVID-19 and Cancer Consortium (CCC19)							
***First Name and Middle Initial(s)**	***Last Name**	***Suffix (eg, Jr, III)**	Academic Degrees	Institution	Location (city, state/province, country)	Role or Contribution, eg, chair, principal investigator	Group (if more than 1 Group listed in the byline) and/or Subgroup (eg, Steering Committee)
Balazs	Halmos		MD	Albert Einstein College of Medicine, Montefiore Medical Center	Bronx, NY	Participating site Co-principal Investigator	CCC19
Amit K.	Verma		MBBS	Albert Einstein College of Medicine, Montefiore Medical Center	Bronx, NY	Participating site Co-principal Investigator	CCC19
Benjamin A.	Gartrell		MD	Albert Einstein College of Medicine, Montefiore Medical Center	Bronx, NY	Participating site Co-Investigator	CCC19
Sanjay	Goel		MBBS	Albert Einstein College of Medicine, Montefiore Medical Center	Bronx, NY	Participating site Co-Investigator	CCC19
Nitin	Ohri		MD	Albert Einstein College of Medicine, Montefiore Medical Center	Bronx, NY	Participating site Co-Investigator	CCC19
R.Alejandro	Sica		MD	Albert Einstein College of Medicine, Montefiore Medical Center	Bronx, NY	Participating site Co-Investigator	CCC19
Astha	Thakkar		MD	Albert Einstein College of Medicine, Montefiore Medical Center	Bronx, NY	Participating site Co-Investigator	CCC19
Keith E.	Stockerl-Goldstein		MD	Alvin J. Siteman Cancer Center at Washington University School of Medicine and Barnes-Jewish HosParticipating site principal Investigatortal	St. Louis, MO	Participating site principal Investigator	CCC19

*Indicates required information. Only first name, last name, and suffix will appear in PubMed.

*First Name and Middle Initial(s)	*Last Name	*Suffix (eg, Jr, III)	Academic Degrees	Institution	Location (city, state/province, country)	Role or Contribution, eg, chair, principal investigator	Group (if more than 1 Group listed in the byline) and/or Subgroup (eg, Steering Committee)
Omar	Butt		MD, PhD	Alvin J. Siteman Cancer Center at Washington University School of Medicine and Barnes-Jewish HosParticipating site principal Investigatortal	St. Louis, MO	Participating site Co-Investigator	CCC19
Jian L.	Campian		MD, PhD	Alvin J. Siteman Cancer Center at Washington University School of Medicine and Barnes-Jewish HosParticipating site principal Investigatortal	St. Louis, MO	Participating site Co-Investigator	CCC19
Mark A.	Fiala		MSW	Alvin J. Siteman Cancer Center at Washington University School of Medicine and Barnes-Jewish HosParticipating site principal Investigatortal	St. Louis, MO	Participating site Co-Investigator	CCC19
Jeffrey P.	Henderson		MD, PhD	Alvin J. Siteman Cancer Center at Washington University School of Medicine and Barnes-Jewish HosParticipating site principal Investigatortal	St. Louis, MO	Participating site Co-Investigator	CCC19
Ryan	Monahan		MBA	Alvin J. Siteman Cancer Center at Washington University School of Medicine and Barnes-Jewish HosParticipating site principal Investigatortal	St. Louis, MO	Participating site Co-Investigator	CCC19
Alice Y.	Zhou		MD, PhD	Alvin J. Siteman Cancer Center at Washington University School of Medicine and Barnes-Jewish HosParticipating site principal Investigatortal	St. Louis, MO	Participating site Co-Investigator	CCC19

Supplemental Online Content: Nonauthor Collaborators

*Indicates required information. Only first name, last name, and suffix will appear in PubMed.

***First Name and Middle Initial(s)**	***Last Name**	***Suffix (eg, Jr, III)**	Academic Degrees	Institution	Location (city, state/province, country)	Role or Contribution, eg, chair, principal investigator	Group (if more than 1 Group listed in the byline) and/or Subgroup (eg, Steering Committee)
Michael A.	Thompson		MD, PhD, FASCO	Aurora Cancer Care, Advocate Aurora Health	Milwaukee, WI	Participating site principal Investigator	CCC19
Pamela	Bohachek		RN	Aurora Cancer Care, Advocate Aurora Health	Milwaukee, WI	Participating site Co-Investigator	CCC19
Daniel	Mundt		MD	Aurora Cancer Care, Advocate Aurora Health	Milwaukee, WI	Participating site Co-Investigator	CCC19
Mitrianna	Streckfuss		MPH	Aurora Cancer Care, Advocate Aurora Health	Milwaukee, WI	Participating site Co-Investigator	CCC19
Eyob	Tadesse		MD	Aurora Cancer Care, Advocate Aurora Health	Milwaukee, WI	Participating site Co-Investigator	CCC19
Philip E.	Lammers		MD, MSCI	Baptist Cancer Center	Memphis, TN	Participating site principal Investigator	CCC19
Orestis A.	Panagiotou		MD,PhD	Brown University and Lifespan Cancer Institute	Providence, RI	Participating site principal Investigator	CCC19
Pamela C.	Egan		MD	Brown University and Lifespan Cancer Institute	Providence, RI	Participating site Co-Investigator	CCC19
Dimitrios	Farmakiotis		MD, FACP, FIDSA	Brown University and Lifespan Cancer Institute	Providence, RI	Participating site Co-Investigator	CCC19
Hina	Khan		MD	Brown University and Lifespan Cancer Institute	Providence, RI	Participating site Co-Investigator	CCC19
Adam J.	Olszewski		MD	Brown University and Lifespan Cancer Institute	Providence, RI	Participating site Co-Investigator	CCC19
Arturo	Loaiza-Bonilla		MD, MSEd, FACP	Cancer Treatment Centers of America	AZ/GA/IL/OK/PA	Participating site principal Investigator	CCC19
Salvatore A.	Del Prete		MD	Carl & Dorothy Bennett Cancer Center at Stamford HosParticipating site principal Investigatortal	Stamford, CT	Participating site principal Investigator	CCC19

*Indicates required information. Only first name, last name, and suffix will appear in PubMed.

*First Name and Middle Initial(s)	*Last Name	*Suffix (eg, Jr, III)	Academic Degrees	Institution	Location (city, state/province, country)	Role or Contribution, eg, chair, principal investigator	Group (if more than 1 Group listed in the byline) and/or Subgroup (eg, Steering Committee)
Michael H.	Bar		MD,FACP	Carl & Dorothy Bennett Cancer Center at Stamford HosParticipating site principal Investigatortal	Stamford, CT	Participating site Co-Investigator	CCC19
Anthony P.	Gulati		MD	Carl & Dorothy Bennett Cancer Center at Stamford HosParticipating site principal Investigatortal	Stamford, CT	Participating site Co-Investigator	CCC19
K. M.	Steve Lo		MD	Carl & Dorothy Bennett Cancer Center at Stamford HosParticipating site principal Investigatortal	Stamford, CT	Participating site Co-Investigator	CCC19
Suzanne J.	Rose		MS, PhD, CCRC, FACRP	Carl & Dorothy Bennett Cancer Center at Stamford HosParticipating site principal Investigatortal	Stamford, CT	Participating site Co-Investigator	CCC19
Jamie	Stratton		MD	Carl & Dorothy Bennett Cancer Center at Stamford HosParticipating site principal Investigatortal	Stamford, CT	Participating site Co-Investigator	CCC19
Paul L.	Weinstein		MD	Carl & Dorothy Bennett Cancer Center at Stamford HosParticipating site principal Investigatortal	Stamford, CT	Participating site Co-Investigator	CCC19
Paolo F.	Caimi		MD	Case Comprehensive Cancer Center at Case Western Reserve University/University HosParticipating site principal Investigatortals	Cleveland, OH	Participating site principal Investigator	CCC19

*Indicates required information. Only first name, last name, and suffix will appear in PubMed.

*First Name and Middle Initial(s)	*Last Name	*Suffix (eg, Jr, III)	Academic Degrees	Institution	Location (city, state/province, country)	Role or Contribution, eg, chair, principal investigator	Group (if more than 1 Group listed in the byline) and/or Subgroup (eg, Steering Committee)
Jill S.	Barnholtz-Sloan		PhD	Case Comprehensive Cancer Center at Case Western Reserve University/University HosParticipating site principal Investigatortals	Cleveland, OH	Participating site Co-Investigator	CCC19
Jorge A.	Garcia		MD, FACP	Case Comprehensive Cancer Center at Case Western Reserve University/University HosParticipating site principal Investigatortals	Cleveland, OH	Participating site Co-Investigator	CCC19
John M.	Nakayama		MD	Case Comprehensive Cancer Center at Case Western Reserve University/University HosParticipating site principal Investigatortals	Cleveland, OH	Participating site Co-Investigator	CCC19
Shilpa	Gupta		MD	Cleveland Clinic	Cleveland, OH	Participating site principal Investigator	CCC19
Nathan A.	Pennell		MD, PhD, FASCO	Cleveland Clinic	Cleveland, OH	Participating site Co-Investigator	CCC19
Manmeet S.	Ahluwalia		MD, FACP	Cleveland Clinic	Cleveland, OH	Participating site Co-Investigator	CCC19
Scott J.	Dawsey		MD	Cleveland Clinic	Cleveland, OH	Participating site Co-Investigator	CCC19
Christopher A.	Lemmon		MD	Cleveland Clinic	Cleveland, OH	Participating site Co-Investigator	CCC19
Amanda	Nizam		MD	Cleveland Clinic	Cleveland, OH	Participating site Co-Investigator	CCC19
Claire	Hoppenot		MD	Dan L Duncan Comprehensive Cancer Center at Baylor College of Medicine	Houston, TX	Participating site Co-principal Investigator	CCC19

*Indicates required information. Only first name, last name, and suffix will appear in PubMed.

*First Name and Middle Initial(s)	*Last Name	*Suffix (eg, Jr, III)	Academic Degrees	Institution	Location (city, state/province, country)	Role or Contribution, eg, chair, principal investigator	Group (if more than 1 Group listed in the byline) and/or Subgroup (eg, Steering Committee)
Ang	Li		MD, MS	Dan L Duncan Comprehensive Cancer Center at Baylor College of Medicine	Houston, TX	Participating site Co-principal Investigator	CCC19
Toni K.	Choueiri		MD	Dana-Farber Cancer Institute	Boston, MA	Participating site principal Investigator	CCC19
Ziad	Bakouny		MD, MSc	Dana-Farber Cancer Institute	Boston, MA	Participating site Co-Investigator	CCC19
Gabrielle	Bouchard		BS	Dana-Farber Cancer Institute	Boston, MA	Participating site Co-Investigator	CCC19
Fiona J.	Busser		BA	Dana-Farber Cancer Institute	Boston, MA	Participating site Co-Investigator	CCC19
Jean M.	Connors		MD	Dana-Farber Cancer Institute	Boston, MA	Participating site Co-Investigator	CCC19
Catherine R.	Curran		BA	Dana-Farber Cancer Institute	Boston, MA	Participating site Co-Investigator	CCC19
George D.	Demetri		MD, FASCO	Dana-Farber Cancer Institute	Boston, MA	Participating site Co-Investigator	CCC19
Antonio	Giordano		MD, PhD	Dana-Farber Cancer Institute	Boston, MA	Participating site Co-Investigator	CCC19
Kaitlin	Kelleher		BA	Dana-Farber Cancer Institute	Boston, MA	Participating site Co-Investigator	CCC19
Anju	Nohria		MD	Dana-Farber Cancer Institute	Boston, MA	Participating site Co-Investigator	CCC19
Andrew	Schmidt		MD	Dana-Farber Cancer Institute	Boston, MA	Participating site Co-Investigator	CCC19
Grace	Shaw		BA	Dana-Farber Cancer Institute	Boston, MA	Participating site Co-Investigator	CCC19
Eli	Van Allen		MD	Dana-Farber Cancer Institute	Boston, MA	Participating site Co-Investigator	CCC19
Pier	Vitale			Dana-Farber Cancer Institute	Boston, MA	Participating site Co-Investigator	CCC19

*Indicates required information. Only first name, last name, and suffix will appear in PubMed.

***First Name and Middle Initial(s)**	***Last Name**	***Suffix (eg, Jr, III)**	Academic Degrees	Institution	Location (city, state/province, country)	Role or Contribution, eg, chair, principal investigator	Group (if more than 1 Group listed in the byline) and/or Subgroup (eg, Steering Committee)
Vincent	Xu		MD	Dana-Farber Cancer Institute	Boston, MA	Participating site Co-Investigator	CCC19
Rebecca L.	Zon		MD	Dana-Farber Cancer Institute	Boston, MA	Participating site Co-Investigator	CCC19
Tian	Zhang		MD, MHS	Duke Cancer Institute at Duke University Medical Center	Durham, NC	Participating site principal Investigator	CCC19
Susan	Halabi		PhD, FASCO	Duke Cancer Institute at Duke University Medical Center	Durham, NC	Participating site Co-Investigator	CCC19
John C.	Leighton	Jr.	MD, FACP	Einstein Healthcare Network	Philadelphia, PA	Participating site principal Investigator	CCC19
Gary H.	Lyman		MD, MPH, FASCO, FRCP	Fred Hutchinson Cancer Research Center/University of Washington/Seattle Cancer Care Alliance	Seattle, WA	Participating site principal Investigator	CCC19
Jerome J.	Graber		MD, MPH	Fred Hutchinson Cancer Research Center/University of Washington/Seattle Cancer Care Alliance	Seattle, WA	Participating site Co-Investigator	CCC19
Petros	Grivas		MD, PhD	Fred Hutchinson Cancer Research Center/University of Washington/Seattle Cancer Care Alliance	Seattle, WA	Participating site Co-Investigator	CCC19
Ali Raza	Khaki		MD	Fred Hutchinson Cancer Research Center/University of Washington/Seattle Cancer Care Alliance	Seattle, WA	Participating site Co-Investigator	CCC19
Elizabeth T.	Loggers		MD,PhD	Fred Hutchinson Cancer Research Center/University of Washington/Seattle Cancer Care Alliance	Seattle, WA	Participating site Co-Investigator	CCC19

*Indicates required information. Only first name, last name, and suffix will appear in PubMed.

*First Name and Middle Initial(s)	*Last Name	*Suffix (eg, Jr, III)	Academic Degrees	Institution	Location (city, state/province, country)	Role or Contribution, eg, chair, principal investigator	Group (if more than 1 Group listed in the byline) and/or Subgroup (eg, Steering Committee)
Ryan C.	Lynch		MD	Fred Hutchinson Cancer Research Center/University of Washington/Seattle Cancer Care Alliance	Seattle, WA	Participating site Co-Investigator	CCC19
Elizabeth S.	Nakasone		MD,PhD	Fred Hutchinson Cancer Research Center/University of Washington/Seattle Cancer Care Alliance	Seattle, WA	Participating site Co-Investigator	CCC19
Michael T.	Schweizer		MD	Fred Hutchinson Cancer Research Center/University of Washington/Seattle Cancer Care Alliance	Seattle, WA	Participating site Co-Investigator	CCC19
Lisa	Tachiki		MD	Fred Hutchinson Cancer Research Center/University of Washington/Seattle Cancer Care Alliance	Seattle, WA	Participating site Co-Investigator	CCC19
Shaveta	Vinayak		MD,MS	Fred Hutchinson Cancer Research Center/University of Washington/Seattle Cancer Care Alliance	Seattle, WA	Participating site Co-Investigator	CCC19
Michael J.	Wagner		MD	Fred Hutchinson Cancer Research Center/University of Washington/Seattle Cancer Care Alliance	Seattle, WA	Participating site Co-Investigator	CCC19
Albert	Yeh		MD	Fred Hutchinson Cancer Research Center/University of Washington/Seattle Cancer Care Alliance	Seattle, WA	Participating site Co-Investigator	CCC19
Na Tosha N.	Gatson		MD, PhD, FAAN	Geisinger Health System	PA, USA	Participating site principal Investigator	CCC19

*Indicates required information. Only first name, last name, and suffix will appear in PubMed.

*First Name and Middle Initial(s)	*Last Name	*Suffix (eg, Jr, III)	Academic Degrees	Institution	Location (city, state/province, country)	Role or Contribution, eg, chair, principal investigator	Group (if more than 1 Group listed in the byline) and/or Subgroup (eg, Steering Committee)
Sharad	Goyal		MD	George Washington University	Washington, DC	Participating site Co-principal Investigator	CCC19
Minh-Phuong	Huynh-Le		MD, MAS	George Washington University	Washington, DC	Participating site Co-principal Investigator	CCC19
Lori J.	Rosenstein		MD	Gundersen Health System	WI, USA	Participating site principal Investigator	CCC19
Peter Paul	Yu		MD, FACP, FASCO	Hartford HealthCare Cancer Institute	Hartford, CT	Participating site principal Investigator	CCC19
Jessica M.	Clement		MD	Hartford HealthCare Cancer Institute	Hartford, CT	Participating site Co-Investigator	CCC19
Ahmad	Daher		MD	Hartford HealthCare Cancer Institute	Hartford, CT	Participating site Co-Investigator	CCC19
Mark	Dailey		MD	Hartford HealthCare Cancer Institute	Hartford, CT	Participating site Co-Investigator	CCC19
Rawad	Elias		MD	Hartford HealthCare Cancer Institute	Hartford, CT	Participating site Co-Investigator	CCC19
Asha	Jayaraj		MD	Hartford HealthCare Cancer Institute	Hartford, CT	Participating site Co-Investigator	CCC19
Emily	Hsu		MD	Hartford HealthCare Cancer Institute	Hartford, CT	Participating site Co-Investigator	CCC19
Alvaro G.	Menendez		MD	Hartford HealthCare Cancer Institute	Hartford, CT	Participating site Co-Investigator	CCC19
Joerg	Rathmann		MD	Hartford HealthCare Cancer Institute	Hartford, CT	Participating site Co-Investigator	CCC19
Oscar	Serrano		MD	Hartford HealthCare Cancer Institute	Hartford, CT	Participating site Co-Investigator	CCC19
Clara	Hwang		MD	Henry Ford Cancer Institute, Henry Ford HosParticipating site principal Investigatortal	Detroit, MI	Participating site principal Investigator	CCC19

*Indicates required information. Only first name, last name, and suffix will appear in PubMed.

*First Name and Middle Initial(s)	*Last Name	*Suffix (eg, Jr, III)	Academic Degrees	Institution	Location (city, state/province, country)	Role or Contribution, eg, chair, principal investigator	Group (if more than 1 Group listed in the byline) and/or Subgroup (eg, Steering Committee)
Shirish M.	Gadgeel		MD	Henry Ford Cancer Institute, Henry Ford HosParticipating site principal Investigatortal	Detroit, MI	Participating site Co-Investigator	CCC19
Sunny R K	Singh		MD	Henry Ford Cancer Institute, Henry Ford HosParticipating site principal Investigatortal	Detroit, MI	Participating site Co-Investigator	CCC19
Jessica E.	Hawley		MD	Herbert Irving Comprehensive Cancer Center at Columbia University		Participating site Co-principal Investigator	CCC19
Dawn	Hershman		MD, MS, FASCO	Herbert Irving Comprehensive Cancer Center at Columbia University		Participating site Co-principal Investigator	CCC19
Melissa K.	Accordino		MD, MS	Herbert Irving Comprehensive Cancer Center at Columbia University		Participating site Co-Investigator	CCC19
Divaya	Bhutani		MD	Herbert Irving Comprehensive Cancer Center at Columbia University		Participating site Co-Investigator	CCC19
Gary K.	Schwartz		MD	Herbert Irving Comprehensive Cancer Center at Columbia University		Participating site Co-Investigator	CCC19
Daniel Y.	Reuben		MD, MS	Hollings Cancer Center at the Medical University of South Carolina	Charleston, SC	Participating site principal Investigator	CCC19
Mariam	Alexander		MD, PhD	Hollings Cancer Center at the Medical University of South Carolina	Charleston, SC	Participating site Co-Investigator	CCC19
Sarah	Mushtaq		MD	Hollings Cancer Center at the Medical University of South Carolina	Charleston, SC	Participating site Co-Investigator	CCC19

Supplemental Online Content: Nonauthor Collaborators

*Indicates required information. Only first name, last name, and suffix will appear in PubMed.

*First Name and Middle Initial(s)	*Last Name	*Suffix (eg, Jr, III)	Academic Degrees	Institution	Location (city, state/province, country)	Role or Contribution, eg, chair, principal investigator	Group (if more than 1 Group listed in the byline) and/or Subgroup (eg, Steering Committee)
Eric H.	Bernicker		MD	Houston Methodist Cancer Center	Houston, TX	Participating site principal Investigator	CCC19
John	Deeken		MD	Inova Schar Cancer Institute	Fairfax, VA	Participating site principal Investigator	CCC19
Danielle	Shafer		DO	Inova Schar Cancer Institute	Fairfax, VA	Participating site Co-Investigator	CCC19
Mark A.	Lewis		MD	Intermountain Health Care	Salt Lake City, UT	Participating site Co-principal Investigator	CCC19
Terence D.	Rhodes		MD, PhD	Intermountain Health Care	Salt Lake City, UT	Participating site Co-principal Investigator	CCC19
David M.	Gill		MD	Intermountain Health Care	Salt Lake City, UT	Participating site Co-Investigator	CCC19
Clarke A.	Low		MD	Intermountain Health Care	Salt Lake City, UT	Participating site Co-Investigator	CCC19
Sandeep H.	Mashru		MD	Kaiser Permanente Northwest	OR/WA	Participating site principal Investigator	CCC19
Abdul-Hai	Mansoor		MD	Kaiser Permanente Northwest	OR/WA	Participating site Co-Investigator	CCC19
Howard A.	Zaren		MD, FACS	Lewis Cancer & Research Pavilion @ St. Joseph's/Candler	Savannah, GA	Participating site principal Investigator	CCC19
Stephanie J.	Smith		RN, MSN, OCN	Lewis Cancer & Research Pavilion @ St. Joseph's/Candler	Savannah, GA	Participating site Co-Investigator	CCC19
Gayathri	Nagaraj		MD	Loma Linda University Cancer Center	Loma Linda, CA	Participating site principal Investigator	CCC19
Mojtaba	Akhtari		MD	Loma Linda University Cancer Center	Loma Linda, CA	Participating site Co-Investigator	CCC19
Eric	Lau		DO	Loma Linda University Cancer Center	Loma Linda, CA	Participating site Co-Investigator	CCC19
Mark E.	Reeves		MD, PhD	Loma Linda University Cancer Center	Loma Linda, CA	Participating site Co-Investigator	CCC19

*Indicates required information. Only first name, last name, and suffix will appear in PubMed.

***First Name and Middle Initial(s)**	***Last Name**	***Suffix (eg, Jr, III)**	Academic Degrees	Institution	Location (city, state/province, country)	Role or Contribution, eg, chair, principal investigator	Group (if more than 1 Group listed in the byline) and/or Subgroup (eg, Steering Committee)
Stephanie	Berg		DO	Loyola University Medical Center	Maywood, IL	Participating site principal Investigator	CCC19
Destry	Elms		MD	Loyola University Medical Center	Maywood, IL	Participating site Co-Investigator	CCC19
Alicia K.	Morgans		MD, MPH	Lurie Cancer Center at Northwestern University	Chicago, IL	Participating site Co-principal Investigator	CCC19
Firas H.	Wehbe		MD, PhD	Lurie Cancer Center at Northwestern University	Chicago, IL	Participating site Co-principal Investigator	CCC19
Jessica	Altman		MD	Lurie Cancer Center at Northwestern University	Chicago, IL	Participating site Co-Investigator	CCC19
Michael	Gurley		BA	Lurie Cancer Center at Northwestern University	Chicago, IL	Participating site Co-Investigator	CCC19
Mary F.	Mulcahy		MD	Lurie Cancer Center at Northwestern University	Chicago, IL	Participating site Co-Investigator	CCC19
Eric B.	Durbin		DrPH, MS	Markey Cancer Center at the University of Kentucky	Lexington, KY	Participating site principal Investigator	CCC19
Amit A.	Kulkarni		MD	Masonic Cancer Center at the University of Minnesota	Minneapolis, MN,	Participating site principal Investigator	CCC19
Heather H.	Nelson		PhD, MPH	Masonic Cancer Center at the University of Minnesota	Minneapolis, MN,	Participating site Co-Investigator	CCC19
Zohar	Sachs		MD, PhD	Masonic Cancer Center at the University of Minnesota	Minneapolis, MN,	Participating site Co-Investigator	CCC19
Surbhi	Shah		MD	Masonic Cancer Center at the University of Minnesota	Minneapolis, MN,	Participating site Co-Investigator	CCC19
Rachel P.	Rosovsky		MD, MPH	Massachusetts General HosParticipating site principal Investigatortal Cancer Center	Boston, MA	Participating site Co-principal Investigator	CCC19
Kerry	Reynolds		MD	Massachusetts General HosParticipating site principal Investigatortal Cancer Center	Boston, MA	Participating site Co-principal Investigator	CCC19

Supplemental Online Content: Nonauthor Collaborators

*Indicates required information. Only first name, last name, and suffix will appear in PubMed.

*First Name and Middle Initial(s)	*Last Name	*Suffix (eg, Jr, III)	Academic Degrees	Institution	Location (city, state/province, country)	Role or Contribution, eg, chair, principal investigator	Group (if more than 1 Group listed in the byline) and/or Subgroup (eg, Steering Committee)
Aditya	Bardia		MD	Massachusetts General HosParticipating site principal Investigatortal Cancer Center	Boston, MA	Participating site Co-Investigator	CCC19
Genevieve	Boland		MD, PhD, FACS	Massachusetts General HosParticipating site principal Investigatortal Cancer Center	Boston, MA	Participating site Co-Investigator	CCC19
Justin	Gainor		MD	Massachusetts General HosParticipating site principal Investigatortal Cancer Center	Boston, MA	Participating site Co-Investigator	CCC19
Leyre	Zubiri		MD, PhD	Massachusetts General HosParticipating site principal Investigatortal Cancer Center	Boston, MA	Participating site Co-Investigator	CCC19
Thorvardur R.	Halfdanarson		MD	Mayo Clinic	AZ/FL/MN	Participating site principal Investigator	CCC19
Tanios	Bekaii-Saab		MD	Mayo Clinic	AZ/FL/MN	Participating site Co-Investigator	CCC19
Aakash	Desai		MD, MPH	Mayo Clinic	AZ/FL/MN	Participating site Co-Investigator	CCC19
Zhuoer	Xie		MD, MS	Mayo Clinic	AZ/FL/MN	Participating site Co-Investigator	CCC19
Ruben A.	Mesa		MD, FACP	Mays Cancer Center at UT Health San Antonio MD Anderson Cancer Center	San Antonio, TX	Participating site principal Investigator	CCC19
Mark	Bonnen		MD	Mays Cancer Center at UT Health San Antonio MD Anderson Cancer Center	San Antonio, TX	Participating site Co-Investigator	CCC19
Daruka	Mahadevan		MD, PhD	Mays Cancer Center at UT Health San Antonio MD Anderson Cancer Center	San Antonio, TX	Participating site Co-Investigator	CCC19

*Indicates required information. Only first name, last name, and suffix will appear in PubMed.

*First Name and Middle Initial(s)	*Last Name	*Suffix (eg, Jr, III)	Academic Degrees	Institution	Location (city, state/province, country)	Role or Contribution, eg, chair, principal investigator	Group (if more than 1 Group listed in the byline) and/or Subgroup (eg, Steering Committee)
Amelie G.	Ramirez		DrPH, MPH	Mays Cancer Center at UT Health San Antonio MD Anderson Cancer Center	San Antonio, TX	Participating site Co-Investigator	CCC19
Mary	Salazar		ANP	Mays Cancer Center at UT Health San Antonio MD Anderson Cancer Center	San Antonio, TX	Participating site Co-Investigator	CCC19
Dimpy P.	Shah		MD, PhD	Mays Cancer Center at UT Health San Antonio MD Anderson Cancer Center	San Antonio, TX	Participating site Co-Investigator	CCC19
Pankil K.	Shah		MD, MSPH	Mays Cancer Center at UT Health San Antonio MD Anderson Cancer Center	San Antonio, TX	Participating site Co-Investigator	CCC19
Bryan	Faller		MD	Missouri Baptist Medical Center	St. Louis, MO	Participating site principal Investigator	CCC19
Rana R.	McKay		MD	Moores Comprehensive Cancer Center at the University of California	San Diego, La Jolla, CA	Participating site principal Investigator	CCC19
Archana	Ajmera		MSN, ANP-BC, AOCNP	Moores Comprehensive Cancer Center at the University of California	San Diego, La Jolla, CA	Participating site Co-Investigator	CCC19
Sharon S.	Brouha		MD, MPH	Moores Comprehensive Cancer Center at the University of California	San Diego, La Jolla, CA	Participating site Co-Investigator	CCC19
Angelo	Cabal		BS	Moores Comprehensive Cancer Center at the University of California	San Diego, La Jolla, CA	Participating site Co-Investigator	CCC19
Albert	Hsiao		MD, PhD	Moores Comprehensive Cancer Center at the University of California	San Diego, La Jolla, CA	Participating site Co-Investigator	CCC19

Supplemental Online Content: Nonauthor Collaborators

*Indicates required information. Only first name, last name, and suffix will appear in PubMed.

*First Name and Middle Initial(s)	*Last Name	*Suffix (eg, Jr, III)	Academic Degrees	Institution	Location (city, state/province, country)	Role or Contribution, eg, chair, principal investigator	Group (if more than 1 Group listed in the byline) and/or Subgroup (eg, Steering Committee)
Seth	Kligerman		MD	Moores Comprehensive Cancer Center at the University of California	San Diego, La Jolla, CA	Participating site Co-Investigator	CCC19
Justin A.	Shaya		MD	Moores Comprehensive Cancer Center at the University of California	San Diego, La Jolla, CA	Participating site Co-Investigator	CCC19
Lisa B.	Weissmann		MD	Mount Auburn HosParticipating site principal Investigatortal	Cambridge, MA	Participating site principal Investigator	CCC19
Chinmay	Jani		MD	Mount Auburn HosParticipating site principal Investigatortal	Cambridge, MA	Participating site Co-Investigator	CCC19
Carey C.	Thomson		MD, FCCP, MPH	Mount Auburn HosParticipating site principal Investigatortal	Cambridge, MA	Participating site Co-Investigator	CCC19
Jeanna	Knoble		MD	Mount Carmel Health System	Columbus, OH	Participating site principal Investigator	CCC19
Mary Grace	Glace		RN	Mount Carmel Health System	Columbus, OH	Participating site Co-Investigator	CCC19
Cameron	Rink		PhD, MBA	Mount Carmel Health System	Columbus, OH	Participating site Co-Investigator	CCC19
Karen	Stauffer		RN	Mount Carmel Health System	Columbus, OH	Participating site Co-Investigator	CCC19
Rosemary	Zacks		RN	Mount Carmel Health System	Columbus, OH	Participating site Co-Investigator	CCC19
Sibel	Blau		MD	Northwest Medical Specialties	Tacoma, WA	Participating site principal Investigator	CCC19
Monika	Joshi		MD, MRCP	Penn State Health/Penn State Cancer Institute/St. Joseph Cancer Center	PA, USA	Participating site principal Investigator	CCC19
Harry	Menon		DO, MPH	Penn State Health/Penn State Cancer Institute/St. Joseph Cancer Center	PA, USA	Participating site Co-Investigator	CCC19

*Indicates required information. Only first name, last name, and suffix will appear in PubMed.

*First Name and Middle Initial(s)	*Last Name	*Suffix (eg, Jr, III)	Academic Degrees	Institution	Location (city, state/province, country)	Role or Contribution, eg, chair, principal investigator	Group (if more than 1 Group listed in the byline) and/or Subgroup (eg, Steering Committee)
Marc A.	Rovito		MD, FACP	Penn State Health/Penn State Cancer Institute/St. Joseph Cancer Center	PA, USA	Participating site Co-Investigator	CCC19
Elizabeth A.	Griffiths		MD	Roswell Park Comprehensive Cancer Center	Buffalo, NY	Participating site principal Investigator	CCC19
Amro	Elshoury		MBBCh	Roswell Park Comprehensive Cancer Center	Buffalo, NY	Participating site Co-Investigator	CCC19
Salma K.	Jabbour		MD	Rutgers Cancer Institute of New Jersey at Rutgers Biomedical and Health Sciences	New Brunswick, NJ	Participating site principal Investigator	CCC19
Christian F.	Misdary		MD	Rutgers Cancer Institute of New Jersey at Rutgers Biomedical and Health Sciences	New Brunswick, NJ	Participating site Co-Investigator	CCC19
Mansi R.	Shah		MD	Rutgers Cancer Institute of New Jersey at Rutgers Biomedical and Health Sciences	New Brunswick, NJ	Participating site Co-Investigator	CCC19
Babar	Bashir		MD, MS	Sidney Kimmel Cancer Center at Thomas Jefferson University	Philadelphia, PA	Participating site principal Investigator	CCC19
Christopher	McNair		PhD	Sidney Kimmel Cancer Center at Thomas Jefferson University	Philadelphia, PA	Participating site Co-Investigator	CCC19
Sana Z.	Mahmood		BA, BS	Sidney Kimmel Cancer Center at Thomas Jefferson University	Philadelphia, PA	Participating site Co-Investigator	CCC19
Vasil	Mico		BS	Sidney Kimmel Cancer Center at Thomas Jefferson University	Philadelphia, PA	Participating site Co-Investigator	CCC19
Andrea Verghese	Rivera		MD	Sidney Kimmel Cancer Center at Thomas Jefferson University	Philadelphia, PA	Participating site Co-Investigator	CCC19
Daniel B.	Flora		MD, PharmD	St. Elizabeth Healthcare	Edgewood, KY	Participating site principal Investigator	CCC19
Barbara B.	Logan		MS	St. Elizabeth Healthcare	Edgewood, KY	Participating site Co-Investigator	CCC19

Supplemental Online Content: Nonauthor Collaborators

*Indicates required information. Only first name, last name, and suffix will appear in PubMed.

*First Name and Middle Initial(s)	*Last Name	*Suffix (eg, Jr, III)	Academic Degrees	Institution	Location (city, state/province, country)	Role or Contribution, eg, chair, principal investigator	Group (if more than 1 Group listed in the byline) and/or Subgroup (eg, Steering Committee)
Goetz	Kloecker		MD	St. Elizabeth Healthcare	Edgewood, KY	Participating site Co-Investigator	CCC19
Chaitanya	Mandapakala		MD	St. Elizabeth Healthcare	Edgewood, KY	Participating site Co-Investigator	CCC19
Sumit A.	Shah		MD, MPH	Stanford Cancer Institute at Stanford University	Palo Alto, CA	Participating site principal Investigator	CCC19
Elwyn C.	Cabebe		MD	Stanford Cancer Institute at Stanford University	Palo Alto, CA	Participating site Co-Investigator	CCC19
Michael J.	Glover		MD	Stanford Cancer Institute at Stanford University	Palo Alto, CA	Participating site Co-Investigator	CCC19
Alokkumar	Jha		PhD	Stanford Cancer Institute at Stanford University	Palo Alto, CA	Participating site Co-Investigator	CCC19
Lidia	Schapira		MD, FASCO	Stanford Cancer Institute at Stanford University	Palo Alto, CA	Participating site Co-Investigator	CCC19
Julie Tsu-Yu	Wu		MD, PhD	Stanford Cancer Institute at Stanford University	Palo Alto, CA	Participating site Co-Investigator	CCC19
Suki	Subbiah		MD	Stanley S. Scott Cancer Center at LSU Health Sciences Center	New Orleans, LA	Participating site principal Investigator	CCC19
Gilberto de Lima	Lopes	Jr.	MD, MBA, FAMS, FASCO	Sylvester Comprehensive Cancer Center at the University of Miami Miller School of Medicine	Miami, FL	Participating site principal Investigator	CCC19
Sanjay G.	Revankar		MD, FIDSA	The Barbara Ann Karmanos Cancer Institute at Wayne State University School of Medicine	Detroit, MI	Participating site principal Investigator	CCC19
Daniel G.	Stover		MD	The Ohio State University Comprehensive Cancer Center	Columbus, OH	Participating site principal Investigator	CCC19
Daniel	Addison		MD	The Ohio State University Comprehensive Cancer Center	Columbus, OH	Participating site Co-Investigator	CCC19
James L.	Chen		MD	The Ohio State University Comprehensive Cancer Center	Columbus, OH	Participating site Co-Investigator	CCC19

Supplemental Online Content: Nonauthor Collaborators

*Indicates required information. Only first name, last name, and suffix will appear in PubMed.

*First Name and Middle Initial(s)	*Last Name	*Suffix (eg, Jr, III)	Academic Degrees	Institution	Location (city, state/province, country)	Role or Contribution, eg, chair, principal investigator	Group (if more than 1 Group listed in the byline) and/or Subgroup (eg, Steering Committee)
Margaret E.	Gatti-Mays		MD	The Ohio State University Comprehensive Cancer Center	Columbus, OH	Participating site Co-Investigator	CCC19
Sachin R.	Jhawar		MD	The Ohio State University Comprehensive Cancer Center	Columbus, OH	Participating site Co-Investigator	CCC19
Vidhya	Karivedu		MBBS	The Ohio State University Comprehensive Cancer Center	Columbus, OH	Participating site Co-Investigator	CCC19
Maryam B.	Lustberg		MD, MPH	The Ohio State University Comprehensive Cancer Center	Columbus, OH	Participating site Co-Investigator	CCC19
Joshua D.	Palmer		MD	The Ohio State University Comprehensive Cancer Center	Columbus, OH	Participating site Co-Investigator	CCC19
Sarah	Wall		MD	The Ohio State University Comprehensive Cancer Center	Columbus, OH	Participating site Co-Investigator	CCC19
Nicole	Williams		MD	The Ohio State University Comprehensive Cancer Center	Columbus, OH	Participating site Co-Investigator	CCC19
Elizabeth	Wulff-Burchfield		MD	The University of Kansas Cancer Center	Kansas City, KS	Participating site principal Investigator	CCC19
Anup	Kasi		MD, MPH	The University of Kansas Cancer Center	Kansas City, KS	Participating site Co-Investigator	CCC19
Natasha	Edwin		MD	ThedaCare Cancer Care	Appleton, WI	Participating site principal Investigator	CCC19
Melissa	Smits		APC	ThedaCare Cancer Care	Appleton, WI	Participating site Co-Investigator	CCC19
David D.	Chism		MD	Thompson Cancer Survival Center	Knoxville, TN	Participating site principal Investigator	CCC19
Susie	Owenby		RN, CCRP	Thompson Cancer Survival Center	Knoxville, TN	Participating site Co-Investigator	CCC19
Deborah B.	Doroshow		MD, PhD	Tisch Cancer Institute at the Icahn School of Medicine at Mount Sinai	New York, NY	Participating site principal Investigator	CCC19
Matthew D.	Galsky		MD	Tisch Cancer Institute at the Icahn School of Medicine at Mount Sinai	New York, NY	Participating site Co-Investigator	CCC19

Supplemental Online Content: Nonauthor Collaborators

*Indicates required information. Only first name, last name, and suffix will appear in PubMed.

***First Name and Middle Initial(s)**	***Last Name**	***Suffix (eg, Jr, III)**	Academic Degrees	Institution	Location (city, state/province, country)	Role or Contribution, eg, chair, principal investigator	Group (if more than 1 Group listed in the byline) and/or Subgroup (eg, Steering Committee)
Michael	Wotman		MD	Tisch Cancer Institute at the Icahn School of Medicine at Mount Sinai	New York, NY	Participating site Co-Investigator	CCC19
Huili	Zhu		MD	Tisch Cancer Institute at the Icahn School of Medicine at Mount Sinai	New York, NY	Participating site Co-Investigator	CCC19
Julie C.	Fu		MD	Tufts Medical Center Cancer Center	Boston and Stoneham, MA	Participating site principal Investigator	CCC19
Alyson	Fazio		APRN-BC	Tufts Medical Center Cancer Center	Boston and Stoneham, MA	Participating site Co-Investigator	CCC19
Mark H.	Sueyoshi		MD	Tufts Medical Center Cancer Center	Boston and Stoneham, MA	Participating site Co-Investigator	CCC19
Kathryn E.	Huber		MD	Tufts Medical Center Cancer Center	Boston and Stoneham, MA	Participating site Co-Investigator	CCC19
Jonathan	Riess		MD, MS	UC Davis Comprehensive Cancer Center at the University of California at Davis	CA, USA	Participating site principal Investigator	CCC19
Kanishka G.	Patel		MD	UC Davis Comprehensive Cancer Center at the University of California at Davis	CA, USA	Participating site Co-Investigator	CCC19
Samuel M.	Rubinstein		MD	UNC Lineberger Comprehensive Cancer Center	Chapel Hill, NC	Participating site principal Investigator	CCC19
William A.	Wood		MD, MPH	UNC Lineberger Comprehensive Cancer Center	Chapel Hill, NC	Participating site Co-Investigator	CCC19
Christopher	Jensen		MD	UNC Lineberger Comprehensive Cancer Center	Chapel Hill, NC	Participating site Co-Investigator	CCC19
Vaibhav	Kumar		MD	UNC Lineberger Comprehensive Cancer Center	Chapel Hill, NC	Participating site Co-Investigator	CCC19
Trisha M.	Wise-Draper		MD, PhD	University of Cincinnati Cancer Center	Cincinnati, OH	Participating site principal Investigator	CCC19
Syed A.	Ahmad		MD, FACS	University of Cincinnati Cancer Center	Cincinnati, OH	Participating site Co-Investigator	CCC19

*Indicates required information. Only first name, last name, and suffix will appear in PubMed.

*First Name and Middle Initial(s)	*Last Name	*Suffix (eg, Jr, III)	Academic Degrees	Institution	Location (city, state/province, country)	Role or Contribution, eg, chair, principal investigator	Group (if more than 1 Group listed in the byline) and/or Subgroup (eg, Steering Committee)
Punita	Grover		MD	University of Cincinnati Cancer Center	Cincinnati, OH	Participating site Co-Investigator	CCC19
Shuchi	Gulati		MD	University of Cincinnati Cancer Center	Cincinnati, OH	Participating site Co-Investigator	CCC19
Jordan	Kharofa		MD	University of Cincinnati Cancer Center	Cincinnati, OH	Participating site Co-Investigator	CCC19
Tahir	Latif		MBBS, MBA	University of Cincinnati Cancer Center	Cincinnati, OH	Participating site Co-Investigator	CCC19
Michelle	Marcum		MS	University of Cincinnati Cancer Center	Cincinnati, OH	Participating site Co-Investigator	CCC19
Cathleen	Park		MD	University of Cincinnati Cancer Center	Cincinnati, OH	Participating site Co-Investigator	CCC19
Hira G.	Shaikh		MD	University of Cincinnati Cancer Center	Cincinnati, OH	Participating site Co-Investigator	CCC19
Daniel W.	Bowles		MD	University of Colorado Cancer Center	Aurora, CO	Participating site principal Investigator	CCC19
Christoper L.	Geiger		MD	University of Colorado Cancer Center	Aurora, CO	Participating site Co-Investigator	CCC19
Merry-Jennifer	Markham		MD, FACP, FASCO	University of Florida Health Cancer Center	Gainesville, FL	Participating site principal Investigator	CCC19
Rohit	Bishnoi		MD	University of Florida Health Cancer Center	Gainesville, FL	Participating site Co-Investigator	CCC19
Atlantis D.	Russ		MD, PhD	University of Florida Health Cancer Center	Gainesville, FL	Participating site Co-Investigator	CCC19
Chintan	Shah		MD	University of Florida Health Cancer Center	Gainesville, FL	Participating site Co-Investigator	CCC19
Jared D.	Acoba		MD	University of Hawai'i Cancer Center	Honolulu, HI	Participating site principal Investigator	CCC19
Young Soo	Rho		MD, CM	University of Hawai'i Cancer Center	Honolulu, HI	Participating site Co-Investigator	CCC19

*Indicates required information. Only first name, last name, and suffix will appear in PubMed.

*First Name and Middle Initial(s)	*Last Name	*Suffix (eg, Jr, III)	Academic Degrees	Institution	Location (city, state/province, country)	Role or Contribution, eg, chair, principal investigator	Group (if more than 1 Group listed in the byline) and/or Subgroup (eg, Steering Committee)
Lawrence E.	Feldman		MD	University of Illinois HosParticipating site principal Investigatortal & Health Sciences System	Chicago, IL	Participating site principal Investigator	CCC19
Kent F.	Hoskins		MD	University of Illinois HosParticipating site principal Investigatortal & Health Sciences System	Chicago, IL	Participating site principal Investigator	CCC19
Gerald	Gantt	Jr.	MD	University of Illinois HosParticipating site principal Investigatortal & Health Sciences System	Chicago, IL	Participating site Co-Investigator	CCC19
Li C.	Liu		PhD	University of Illinois HosParticipating site principal Investigatortal & Health Sciences System	Chicago, IL	Participating site Co-Investigator	CCC19
Mahir	Khan		MD	University of Illinois HosParticipating site principal Investigatortal & Health Sciences System	Chicago, IL	Participating site Co-Investigator	CCC19
Ryan H.	Nguyen		DO	University of Illinois HosParticipating site principal Investigatortal & Health Sciences System	Chicago, IL	Participating site Co-Investigator	CCC19
Mary	Pasquinelli		APN, DNP	University of Illinois HosParticipating site principal Investigatortal & Health Sciences System	Chicago, IL	Participating site Co-Investigator	CCC19

Supplemental Online Content: Nonauthor Collaborators

*Indicates required information. Only first name, last name, and suffix will appear in PubMed.

***First Name and Middle Initial(s)**	***Last Name**	***Suffix (eg, Jr, III)**	Academic Degrees	Institution	Location (city, state/province, country)	Role or Contribution, eg, chair, principal investigator	Group (if more than 1 Group listed in the byline) and/or Subgroup (eg, Steering Committee)
Candice	Schwartz		MD	University of Illinois HosParticipating site principal Investigatortal & Health Sciences System	Chicago, IL	Participating site Co-Investigator	CCC19
Neeta K.	Venepalli		MD, MBA	University of Illinois HosParticipating site principal Investigatortal & Health Sciences System	Chicago, IL	Participating site Co-Investigator	CCC19
Praveen	Vikas		MD	University of Iowa Holden Comprehensive Cancer Center	Iowa City, IA	Participating site principal Investigator	CCC19
Christopher R.	Friese		PhD, RN, AOCN, FAAN	University of Michigan Rogel Cancer Center	Ann Arbor, MI	Participating site principal Investigator	CCC19
Leslie A.	Fecher		MD	University of Michigan Rogel Cancer Center	Ann Arbor, MI,	Participating site principal Investigator	CCC19
Blanche H.	Mavromatis		MD	UPMC Western Maryland	Cumberland, MD	Participating site principal Investigator	CCC19
Ragneel R.	Bijjula		MD	UPMC Western Maryland	Cumberland, MD	Participating site Co-Investigator	CCC19
Qamar U.	Zaman		MD	UPMC Western Maryland	Cumberland, MD	Participating site Co-Investigator	CCC19
Jeremy L.	Warner		MD, MS, FAMIA, FASCO	Vanderbilt-Ingram Cancer Center at Vanderbilt University Medical Center	Nashville, TN	Participating site principal Investigator	CCC19
Alex	Cheng		PhD	Vanderbilt-Ingram Cancer Center at Vanderbilt University Medical Center	Nashville, TN	Participating site Co-Investigator	CCC19
Elizabeth J.	Davis		MD	Vanderbilt-Ingram Cancer Center at Vanderbilt University Medical Center	Nashville, TN	Participating site Co-Investigator	CCC19

Supplemental Online Content: Nonauthor Collaborators

*Indicates required information. Only first name, last name, and suffix will appear in PubMed.

***First Name and Middle Initial(s)**	***Last Name**	***Suffix (eg, Jr, III)**	Academic Degrees	Institution	Location (city, state/province, country)	Role or Contribution, eg, chair, principal investigator	Group (if more than 1 Group listed in the byline) and/or Subgroup (eg, Steering Committee)
Stephany N.	Duda		PhD, MS	Vanderbilt-Ingram Cancer Center at Vanderbilt University Medical Center	Nashville, TN	Participating site Co-Investigator	CCC19
Kyle T.	Enriquez		MSc, BS	Vanderbilt-Ingram Cancer Center at Vanderbilt University Medical Center	Nashville, TN	Participating site Co-Investigator	CCC19
Benjamin	French		PhD	Vanderbilt-Ingram Cancer Center at Vanderbilt University Medical Center	Nashville, TN	Participating site Co-Investigator	CCC19
Erin A.	Gillaspie		MD, MPH	Vanderbilt-Ingram Cancer Center at Vanderbilt University Medical Center	Nashville, TN	Participating site Co-Investigator	CCC19
Cassandra	Hennessy		MS	Vanderbilt-Ingram Cancer Center at Vanderbilt University Medical Center	Nashville, TN	Participating site Co-Investigator	CCC19
Daniel	Hausrath		MD	Vanderbilt-Ingram Cancer Center at Vanderbilt University Medical Center	Nashville, TN	Participating site Co-Investigator	CCC19
Chih-Yuan	Hsu		PhD	Vanderbilt-Ingram Cancer Center at Vanderbilt University Medical Center	Nashville, TN	Participating site Co-Investigator	CCC19
Douglas B.	Johnson		MD, MSCI	Vanderbilt-Ingram Cancer Center at Vanderbilt University Medical Center	Nashville, TN	Participating site Co-Investigator	CCC19
Xuanyi	Li		BA	Vanderbilt-Ingram Cancer Center at Vanderbilt University Medical Center	Nashville, TN	Participating site Co-Investigator	CCC19
Sanjay	Mishra		MS, PhD	Vanderbilt-Ingram Cancer Center at Vanderbilt University Medical Center	Nashville, TN	Participating site Co-Investigator	CCC19

*Indicates required information. Only first name, last name, and suffix will appear in PubMed.

***First Name and Middle Initial(s)**	***Last Name**	***Suffix (eg, Jr, III)**	Academic Degrees	Institution	Location (city, state/province, country)	Role or Contribution, eg, chair, principal investigator	Group (if more than 1 Group listed in the byline) and/or Subgroup (eg, Steering Committee)
Sonya A.	Reid		MD, MPH	Vanderbilt-Ingram Cancer Center at Vanderbilt University Medical Center	Nashville, TN	Participating site Co-Investigator	CCC19
Brian I.	Rini		MD, FACP, FASCO	Vanderbilt-Ingram Cancer Center at Vanderbilt University Medical Center	Nashville, TN	Participating site Co-Investigator	CCC19
David A.	Slosky		MD	Vanderbilt-Ingram Cancer Center at Vanderbilt University Medical Center	Nashville, TN	Participating site Co-Investigator	CCC19
Yu	Shyr		PhD	Vanderbilt-Ingram Cancer Center at Vanderbilt University Medical Center	Nashville, TN	Participating site Co-Investigator	CCC19
Carmen C.	Solorzano		MD, FACS	Vanderbilt-Ingram Cancer Center at Vanderbilt University Medical Center	Nashville, TN	Participating site Co-Investigator	CCC19
Tianyi	Sun		MS	Vanderbilt-Ingram Cancer Center at Vanderbilt University Medical Center	Nashville, TN	Participating site Co-Investigator	CCC19
Matthew D.	Tucker		MD	Vanderbilt-Ingram Cancer Center at Vanderbilt University Medical Center	Nashville, TN	Participating site Co-Investigator	CCC19
Karen	Vega-Luna		MA	Vanderbilt-Ingram Cancer Center at Vanderbilt University Medical Center	Nashville, TN	Participating site Co-Investigator	CCC19
Lucy L.	Wang		BA	Vanderbilt-Ingram Cancer Center at Vanderbilt University Medical Center	Nashville, TN	Participating site Co-Investigator	CCC19
Hagen F.	Kennecke		MD, MHA, FRCPC	Virginia Mason Cancer Institute	Seattle, WA	Participating site principal Investigator	CCC19

Supplemental Online Content: Nonauthor Collaborators

*Indicates required information. Only first name, last name, and suffix will appear in PubMed.

*First Name and Middle Initial(s)	*Last Name	*Suffix (eg, Jr, III)	Academic Degrees	Institution	Location (city, state/province, country)	Role or Contribution, eg, chair, principal investigator	Group (if more than 1 Group listed in the byline) and/or Subgroup (eg, Steering Committee)
David M.	Aboulafia		MD	Virginia Mason Cancer Institute	Seattle, WA	Participating site Co-Investigator	CCC19
Brett A.	Schroeder		MD	Virginia Mason Cancer Institute	Seattle, WA	Participating site Co-Investigator	CCC19
Matthew	Puc		MD	Virtua Health, Marlton	NJ, USA	Participating site principal Investigator	CCC19
Theresa M.	Carducci		MSN, RN, CCRP	Virtua Health, Marlton	NJ, USA	Participating site Co-Investigator	CCC19
Karen J.	Goldsmith		BSN, RN	Virtua Health, Marlton	NJ, USA	Participating site Co-Investigator	CCC19
Susan	Van Loon		RN, CTR, CCRP	Virtua Health, Marlton	NJ, USA	Participating site Co-Investigator	CCC19
Umit	Topaloglu		PhD, FAMIA	Wake Forest Baptist Comprehensive Cancer Center	Winston-Salem, NC	Participating site principal Investigator	CCC19
Saif I.	Alimohamed		MD	Wake Forest Baptist Comprehensive Cancer Center	Winston-Salem, NC	Participating site Co-Investigator	CCC19
Robert L.	Rice		MD, PhD	WellSpan Health	York, PA	Participating site principal Investigator	CCC19
Wilhelmina D.	Cabalona		MD	Wentworth-Douglass HosParticipating site principal Investigatortal	Dover, NH	Participating site principal Investigator	CCC19
Christine	Pilar		BS, CCRC, ACRP-PM	Wentworth-Douglass HosParticipating site principal Investigatortal	Dover, NH	Participating site Co-Investigator	CCC19
Prakash	Peddi		MD	Willis-Knighton Cancer Center	Shreveport, LA	Participating site principal Investigator	CCC19
Lane R.	Rosen		MD	Willis-Knighton Cancer Center	Shreveport, LA	Participating site principal Investigator	CCC19
Briana Barrow	McCollough		BSc, CCRC	Willis-Knighton Cancer Center	Shreveport, LA	Participating site Co-Investigator	CCC19

Supplemental Online Content: Nonauthor Collaborators

*Indicates required information. Only first name, last name, and suffix will appear in PubMed.

***First Name and Middle Initial(s)**	***Last Name**	***Suffix (eg, Jr, III)**	Academic Degrees	Institution	Location (city, state/province, country)	Role or Contribution, eg, chair, principal investigator	Group (if more than 1 Group listed in the byline) and/or Subgroup (eg, Steering Committee)
Mehmet A.	Bilen		MD	Winship Cancer Institute of Emory University	Atlanta, GA	Participating site principal Investigator	CCC19
Deepak	Ravindranathan		MD, MS	Winship Cancer Institute of Emory University	Atlanta, GA	Participating site Co-Investigator	CCC19
Navid	Hafez		MD, MPH	Yale Cancer Center at Yale University School of Medicine	New Haven, CT	Participating site principal Investigator	CCC19
Roy	Herbst		MD, PhD	Yale Cancer Center at Yale University School of Medicine	New Haven, CT	Participating site Co-Investigator	CCC19
Patricia	LoRusso		DO, PhD	Yale Cancer Center at Yale University School of Medicine	New Haven, CT	Participating site Co-Investigator	CCC19
Tyler	Masters		MS	Yale Cancer Center at Yale University School of Medicine	New Haven, CT	Participating site Co-Investigator	CCC19
Catherine	Stratton		BA	Yale Cancer Center at Yale University School of Medicine	New Haven, CT	Participating site Co-Investigator	CCC19
